# Reflections on the 50‐year history of the Federation of Asian and Oceanian Biochemists and Molecular Biologists (FAOBMB)

**DOI:** 10.1002/iub.2679

**Published:** 2022-10-26

**Authors:** Phillip Nagley, Jisnuson Svasti, Akira Kikuchi

**Affiliations:** ^1^ Department of Biochemistry and Molecular Biology Monash University Clayton Victoria Australia; ^2^ Laboratory of Biochemistry Chulabhorn Research Institute Bangkok Thailand; ^3^ Planning & Management Office Centre for Infectious Disease Education and Research (CiDER), Osaka University Suita Japan

**Keywords:** Asia, Biochemistry, Federation, History, Molecular Biology, Oceania

## Abstract

The Federation of Asian and Oceanian Biochemists and Molecular Biologists, Inc. (FAOBMB) celebrates its Golden Jubilee in 2022. Established in August 1972 as a regional grouping of three national societies of biochemists in Australia, India and Japan, it took the name Federation of Asian and Oceanian Biochemists (FAOB). The Federation rapidly grew to encompass another 12 national societies (or groups) of biochemists within 6 years, eventually increasing the number of Constituent Members to 21 by 2014. FAOB soon established regular scientific meetings, including triennial Congresses and annual Symposia; from 1980 FAOB Travel Fellowships enabled regional young scientists to participate in them. In 1992, FAOB was constituted as an Incorporated Association in Victoria, Australia, changing its name 1 year later (yielding the acronym FAOBMB). A printed Newsletter/Bulletin was distributed through each Constituent Society or Group from 1972 to 1999. With the advent of the internet and email in the late 1990s, communication rapidly improved, such that the first webpage of FAOBMB was set up in 1995. From the inception of the Federation, an international journal sponsored by FAOB was foreshadowed but only commenced in 1997, sadly lasting only 6 years. Education in biochemistry and molecular biology became prominent in FAOBMB from the 1990s. In the 21st century, awards to high‐achieving scientists and educationists were introduced, the first being the Young Scientist Awards in 2006. The Fellowships program was extended to young educationists in 2018. FAOB(MB) has been supported by the International Union of Biochemistry (and Molecular Biology) almost its entire history, mostly for support of Congresses, Conferences and Symposia, but also for Young Scientist Programs. The most recent challenge to FAOBMB came with the COVID‐19 pandemic. Executive Committee and the Constituent Members rapidly adapted to virtual communications for their administrative meetings and Education Symposia, and a memorable Congress was held totally on‐line in 2021.

**Abbreviations**
A‐IMBN: Asia‐Pacific International Molecular Biology Network
ASBMB: Australian Society for Biochemistry and Molecular Biology
EC: Executive Committee
FAOB: Federation of Asian and Oceanian Biochemists
FAOBMB: Federation of Asian and Oceanian Biochemists and Molecular Biologists, Inc.
FASBMB: Federation of African Societies for Biochemistry and Molecular Biology
FEBS: Federation of the European Biochemical Societies
IUB: International Union of Biochemistry
IUBMB: International Union of Biochemistry and Molecular Biology
JBMBB: *Journal of Biochemistry, Molecular Biology and Biophysics*
JBS: Japanese Biochemical Society
JSPS: Japanese Society for the Promotion of Science
PABMB: Pan‐American Association for Biochemistry and Molecular Biology
SBC(I): Society of Biological Chemists (India)
YSP: Young Scientist Program

## 1 INTRODUCTION AND OVERVIEW

The Federation of Asian and Oceanian Biochemists and Molecular Biologists, Inc. (FAOBMB) is a federation of national societies of biochemistry and molecular biology in the Asian and Oceanian Region. The primary purpose of FAOBMB is to promote the science of biochemistry and molecular biology, including education, research and technological applications, worldwide and, in particular, in the Asian and Oceanian Region.

FAOBMB came into existence in 1972, under the simpler name of The Federation of Asian and Oceanian Biochemists (FAOB). This was after the International Union of Biochemistry (IUB) had been founded in 1955 and had consolidated its fully international role as the recognised Scientific Union in the field of Biochemistry, representing national biochemical societies across all continents. IUB had established its cycle of triennial Congresses in various cities, initially mostly in Europe. Interestingly, the first such Congress of Biochemistry was held in 1949, in Cambridge, United Kingdom. The first IUB Congress to be held in Asia was the seventh in the series, held in 1967 in Tokyo, Japan. The principles on which FAOB emerged as a regional federation were based on the success of the Federation of the European Biochemical Societies (FEBS) that had been founded in 1964. FEBS organised regular annual Congresses and, through its role in two very successful international research journals, had accumulated considerable earnings.

**FIGURE 1:**
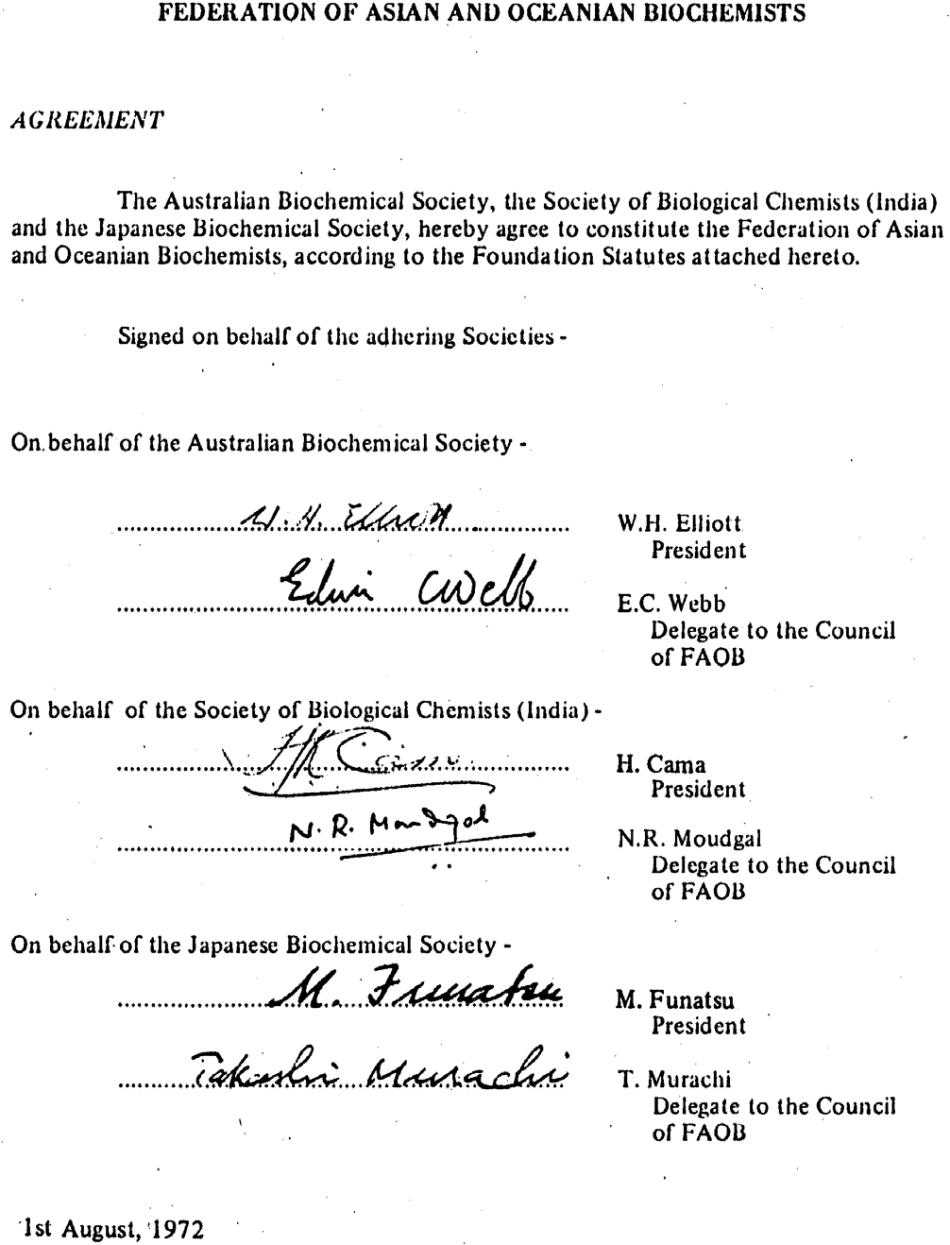
Agreement to found FAOB, dated 1 August 1972, with signatures of both the President and inaugural Delegate to Council of FAOB of each the three founding National Societies of biochemists in Australia, India and Japan

**FIGURE 2:**
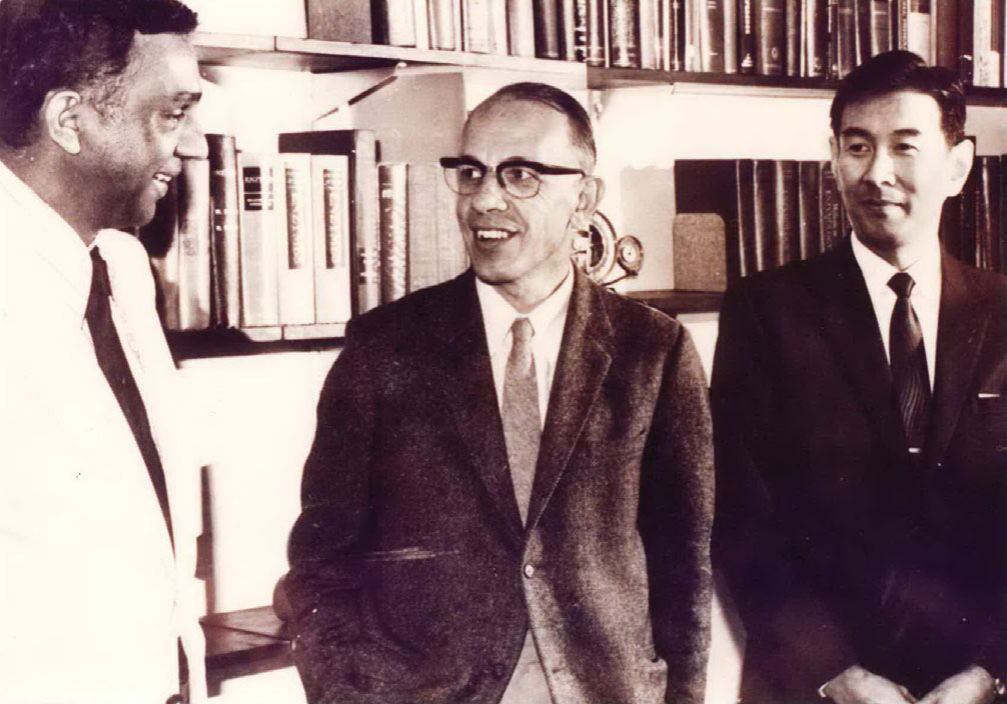
Members of the first Executive Committee of FAOBMB, ca.1972. From left: N. R. Mougdal (India), Edwin C. Webb (Australia), Takashi Murachi (Japan). Photographs were taken in the office of Professor E. C. Webb at Macquarie University in Sydney, on two separate occasions during visits by each of Mougdal and Murachi. Portions of the two photographs were merged to generate this single image. Image created by E. C. Webb and provided to J. Svasti

Against this background, preliminary discussions were held in the late 1960s concerning the desirability of founding a regional federation in the Asia and Oceania region. These discussions were strongly encouraged by William J. Whelan,^1^ an Englishman who was one of the founders of FEBS and its first Secretary General; he had moved to Miami, Florida in 1967 and was the pioneer of the formation of a regional federation of biochemical societies in the Americas. Whelan encouraged leading biochemists in Japan, Australia and India (including, but by no means exclusively, Kunio Yagi from Japan, and Anthony W. Linnane from Australia). In May 1971 in Tokyo, and in February 1972 in Delhi, meetings took place of representatives of the national biochemical societies of Australia, Japan and India to consolidate these proposals. A meeting of the Provisional Committee of FAOB was held in Adelaide in July 1972, which culminated in the signing on 1 August 1972 of a formal agreement to found FAOB. The signatories comprised Edwin C. Webb (Australia), Takashi Murachi (Japan) and N. R. Mougdal (India), together with the then Presidents of the respective national biochemical societies of those countries (Figure 1). That agreement also recognised a set of Statutes that had been drawn up by Webb to manage the operation of the fledgling federation. At its inception, the Executive Committee (EC) of FAOB thereby comprised those three delegates to FAOB Council from Australia, Japan and India, who became the founding President (Webb), Vice‐President (Murachi) and Secretary General/Treasurer (Mougdal) (Figure 2).

During the years that followed, the three founding biochemical societies from which FAOB was initially created were joined by many other national societies (or groups within other broader national scientific societies), as detailed in Table 1. Barely 1 year after FAOB was founded, the number of Constituent Member Societies or Groups had already risen to 10, almost half of the highest such membership ever achieved. Further, by the time of the 20th Anniversary of FAOB in 1992, there were 17 Constituent Members, with only a further four more yet to join in the decades that followed. However, the Hawaii Biochemistry and Molecular Biology Group ceased to become a Constituent Member in 2018, due to shrinking numbers in the group, so that presently FAOBMB consists of 20 Constituent Members.

**TABLE 1:** Constituent Members of the Federation of Asian and Oceanian Biochemists (and Molecular Biologists)^a^

Country	Name of society (or group) as of date of admission	Present name of society (or group)	Date of admission^b^	Size of society (or group)^c^
Australia	Australian Biochemical Society	Australian Society for Biochemistry and Molecular Biology	1 August 1972	Subgroup 3
India	Society of Biological Chemists (India)	Society of Biological Chemists (India)	1 August 1972	Subgroup 4
Japan	Japanese Biochemical Society	Japanese Biochemical Society	1 August 1972	Subgroup 5
China, Hong Kong	Hong Kong Biochemical Association	Hong Kong Society of Biochemistry and Molecular Biology	31 October 1973	Subgroup 1
Indonesia	Indonesian Biochemical Society	Indonesian Society for Biochemistry and Molecular Biology	31 October 1973	Subgroup 2
Korea	The Biochemical Society of the Republic of Korea	Korean Society for Biochemistry and Molecular Biology	31 October 1973	Subgroup 4
New Zealand	New Zealand Biochemical Society	New Zealand Society for Biochemistry and Molecular Biology	31 October 1973	Subgroup 1
Philippines	Philippine Biochemical Society	Philippine Society of Biochemistry and Molecular Biology	31 October 1973	Subgroup 2
Singapore	Singapore Biochemical Society	Singapore Society of Biochemistry and Molecular Biology	31 October 1973	Subgroup 1
Thailand	Biochemical Section, Science Society of Thailand	Biochemistry and Molecular Biology Section, Science Society of Thailand	31 October 1973	Subgroup 2
Pakistan	Pakistan Society of Biochemists	Pakistan Society for Biochemistry and Molecular Biology	26 February 1976	Subgroup 2
Hawaii	Hawaii Biochemical Group	Hawaii Biochemistry and Molecular Biology Group^d^	22 June 1976	Not applicable
China, Taipei	Biochemical Society located in Taipei, China	Society for Biochemistry and Molecular Biology located in Taipei, China^e^	10 December 1976	Subgroup 3
Bangladesh	Bangladesh Biochemical Society	Bangladesh Society for Biochemistry and Molecular Biology	10 April 1977	Subgroup 2
Malaysia	Malaysian Biochemical Society	Malaysian Society for Biochemistry and Molecular Biology	20 September 1978	Subgroup 2
China, Beijing	Chinese Biochemical Society	Chinese Society of Biochemistry and Molecular Biology	11 April 1984	Subgroup 5
Myanmar	Myanmar Biochemists Group	Myanmar Biochemists Group	5 May 1990	Subgroup 1
Vietnam	Biochemists Group of Vietnam—Universities and Pedagogical Institutions	Viet Nam Association of Biochemistry and Molecular Biology	2 September 1993	Subgroup 2
Nepal	Nepal Molecular Biology Society	Nepal Molecular Biology Society	23 September 1995	Subgroup 1
Iran	Biochemical Society of the Islamic Republic of Iran^f^	Biochemical Society of the Islamic Republic of Iran	21 October 2000	Subgroup 2
Sri Lanka	College of Biochemists of Sri Lanka	College of Biochemists of Sri Lanka	1 January 2014	Subgroup 1

^a^ The Federation changed its acronym from FAOB to FAOBMB in 1993, with the official change of its registered name.

^b^ Constituent Members are listed in order of Date of Admission (and listed alphabetically by Country for those admitted on the same date).

^c^ Size is defined according to Appendix I of the Standing Orders of FAOBMB (www.faobmb.com/about‐faobmb/constitution/), as first ratified by FAOBMB Council on 19 August 2020, as follows: Subgroup 1 includes Societies with no more than 100 members; Subgroup 2 includes Societies with more than 100 members but no more than 500 members; Subgroup 3 includes Societies with more than 500 members but no more than 1000 members; Subgroup 4 includes Societies with more than 1000 members but no more than 5000 members; Subgroup 5 includes Societies with more than 5000 members. Members refer to full fee‐paying members, exclusive of student members.

^d^ Membership suspended as of 3 June 2018. The Group size at that date would have corresponded to Subgroup 1.

^e^ This Society refers to itself as the Taiwan Society for Biochemistry and Molecular Biology; however, this name is not officially recognised by FAOBMB.

^f^ This Society is known informally as the Biochemical Society of Iran.

It can be seen from Table 1 that the relative sizes of Constituent Member Societies or Groups cover a wide range, from <100 to more than 5000 full members. This disparity in size (and, therefore, financial and organisational robustness) amongst the Constituent members of FAOB(MB) has been a continuing feature of the Federation, which has tailored its activities and its financial arrangements (such as annual subscriptions) to take these size differences into account. However, there has always been equal opportunity for individual members of the Constituent Societies of FAOB(MB) to participate in the benefits of membership, for example, in terms of each having a single delegate to Council, and access to Travel Fellowships of the Federation.

It is also evident in Table 1 that many Constituent Members have changed their name since joining FAOB, particularly to include Molecular Biology as part of their name alongside Biochemistry. This was part of a worldwide change in such nomenclature, such that IUB had changed its title in 1991 to become the International Union of Biochemistry and Molecular Biology (IUBMB).^2^ Shortly afterwards, at the urging of Bruce A. Stone, Australian delegate to Council, FAOB changed its name in 1993 to the Federation of Asian and Oceanian Biochemists and Molecular Biologists (FAOBMB). Since 1992, FAOB(MB) has been an Incorporated Association registered in the State of Victoria (Australia). FAOBMB is also an active Associate Organization of IUBMB, alongside similar regional federations in Europe (FEBS), Africa (FASBMB) and the Americas (PABMB). IUB began to provide funding for FAOB in 1979, with the strong support of Bill Whelan^1^ who was then General Secretary of IUB; with Whelan's influence as ‘father of the federations’, the category of Associate Organizations of IUB was established in 1982.

**FIGURE 3:**
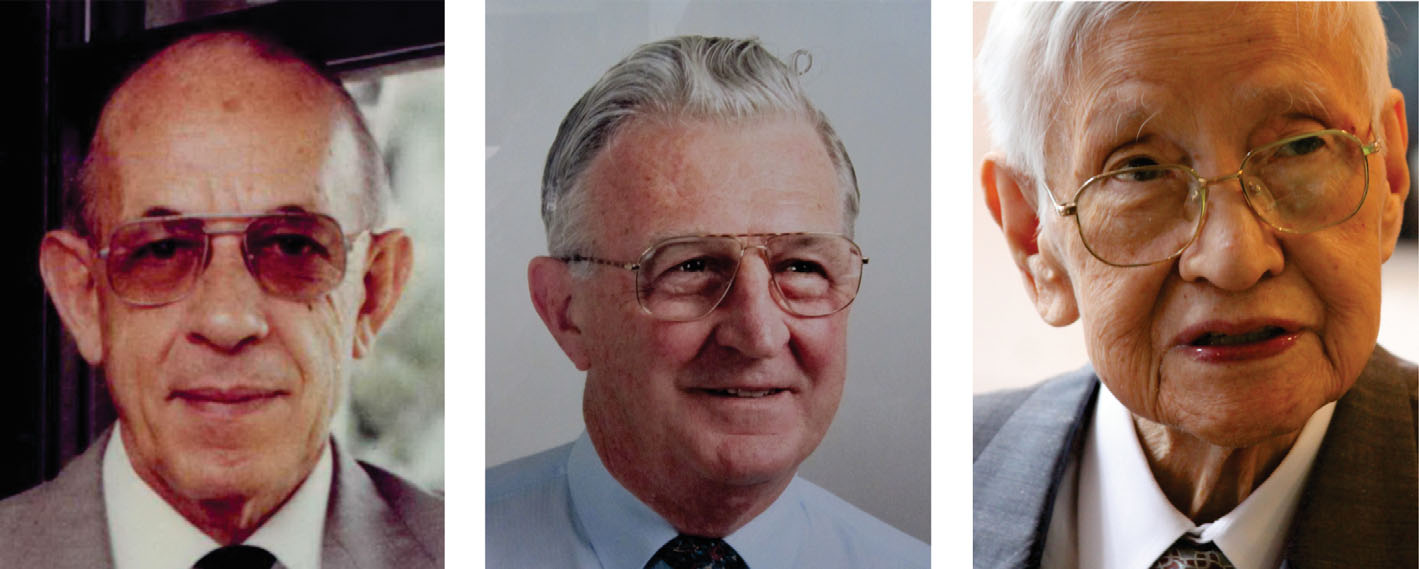
Collage of first three Presidents of FAOBMB. From left: Edwin C. Webb, Anthony W. Linnane, Kazutomo Imahori. Collage prepared by P. Nagley from images provided as follows: Webb, by J. Svasti; Linnane, by P. Nagley: Imahori, by Japanese Biochemical Society

**FIGURE 4:**
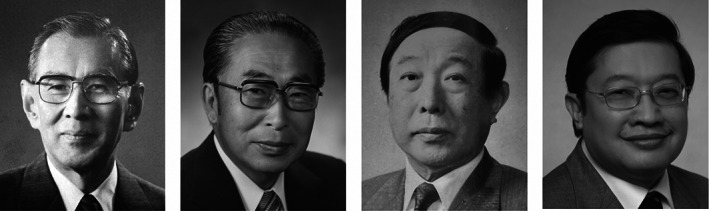
Collage of those after whom FAOBMB Plenary Lectures are named. From left: Takashi Murachi, Osamu Hayaishi, Kunio Yagi, Jisnuson Svasti. Collage prepared by P. Nagley from images provided as follows: Murachi, by J. Svasti; Hayaishi, by Japanese Biochemical Society; Yagi, by Kimie Yagi; Svasti, by J. Svasti

**FIGURE 5:**
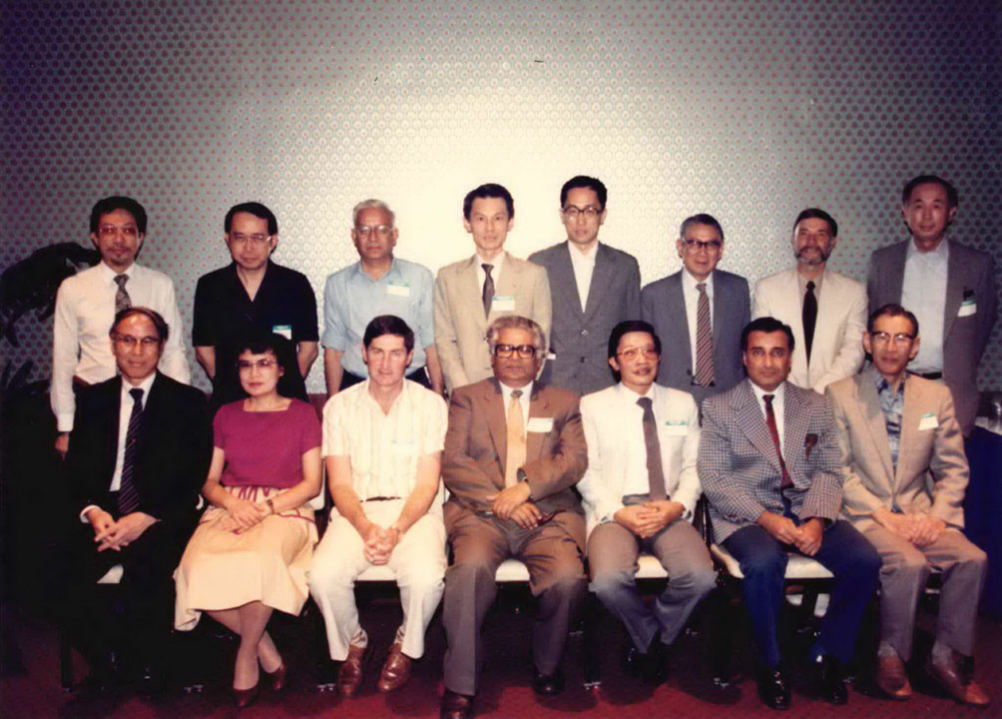
FAOB Executive Committee, Delegates to FAOB Council, and Observers at the 5th FAOB Symposium held in Honolulu, Hawaii, held during 3–6 December 1985. Standing from left: It‐Koon Tan (Observer, Singapore), Kay Hoon Lee (Singapore), Appaji Rao (India), Mu‐chin Tzeng (Observer, Taipei, China), Qi‐Shui Lin (China), Ying‐Lai Wang (Observer, China), Richard J. Guillory (Observer, Hawaii), Yoshito Kaziro (Japan). Sitting, from left: Sang‐Sup Lee (Korea), Lourdes J. Cruz (Philippines), Fyfe L. Bygrave (Secretary General, Australia), Bimal K. Bacchawat (President, India), Jisnuson Svasti (Treasurer, Thailand), Nadhipuram V. Bhagavan (Hawaii), Takashi Murachi (Chair of Fellowships Committee, Japan). Image provided by J. Svasti

**FIGURE 6:**
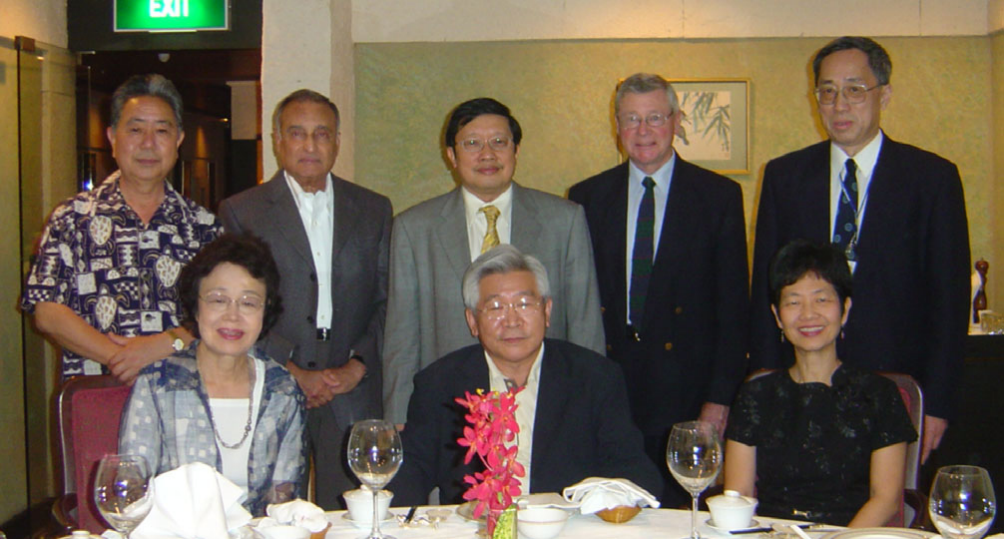
Six Presidents of FAOB(MB) photographed in Bangkok, Thailand at the 17th FAOBMB Symposium in November 2004: Standing from left: Yasuhiro Anraku, Nadhipuram V. Bhagavan, Jisnuson Svasti, William H. Sawyer, Qi‐Shui Lin. Sitting from left: Mrs Naoyo Anraku, Kyung‐soo Hahm, Hoon‐Eng Khoo (Secretary General). Image provided by J. Svasti

**FIGURE 7:**
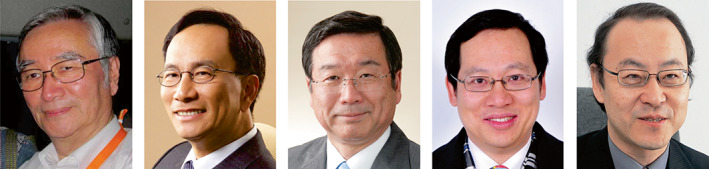
Collage of Presidents 2008‐present. From left: Masamitsu Futai, Andrew. H. J. Wang, Kiyoshi Fukui, Zengyi Chang, Akira Kikuchi. Collage prepared by P. Nagley from FAOBMB Archives; images were provided by each of those depicted

Since its founding in 1972, FAOB(MB) has developed steadily, playing an important catalytic role in stimulating life sciences, technology transfer and entrepreneurship. FAOBMB works closely with both its Constituent Members and IUBMB to advance education, research, and applications of biochemistry and molecular biology. The development and prospering of FAOB(MB) over the past 50 years has taken place under the leadership of a series of Presidents (Table 2). Whilst it has not been possible to compile a single composite image with portrait photographs of each of the 17 Presidents over this period, there is at least one image of each President in the present article, distributed amongst collages of single images and group photographs (Figures 3, 4, 5, 6, 7), as indicated in the rightmost column of Table 2.

**TABLE 2:** Presidents of the Federation of Asian and Oceanian Biochemists (and Molecular Biologists)^a^

Name of president	Country	Dates of presidency^b^	Photograph^c^
Edwin C. Webb	Australia	1972–1974	Figure 3
Anthony W. Linnane	Australia	1975–1977	Figure 3
Kazutomo Imahori	Japan	1978–1980	Figure 3
Osamu Hayaishi	Japan	1981–1983	Figure 4
Bimal K. Bacchawat	India	1984–1986	Figure 5
Takashi Murachi	Japan	1987–1989	Figure 4
Jisnuson Svasti	Thailand	1990–1992	Figure 4
Nadhipuram V. Bhagavan	Hawaii	1993–1995	Figure 6
Yasuhiro Anraku	Japan	1996–1998	Figure 6
William H. Sawyer	Australia	1999–2001	Figure 6
Qi‐Shui Lin	China, Beijing	2002–2004	Figure 6
Kyung‐soo Hahm	Korea	2005–2007	Figure 6
Masamitsu Futai	Japan	2008–2010	Figure 7
Andrew H. J. Wang	China, Taipei	2011–2013	Figure 7
Kiyoshi Fukui	Japan	2014–2016	Figure 7
Zengyi Chang	China, Beijing	2017–2019	Figure 7
Akira Kikuchi	Japan	2020–2022	Figure 7

^a^ The Federation changed its acronym from FAOB to FAOBMB in 1993, with the official change of its registered name.

^b^ According to current Rules of FAOBMB, Presidents serve as President‐Elect for one year before assuming the Presidency (for a 3‐year term), then for a further two years as Past‐President.

^c^ Refers to one of the Figures in this article.

The purpose of this history of FAOB(MB) is to review the development and achievements of the Federation and to consider the challenges it has faced over the past 50 years and those that confront the Federation into the foreseeable future. These aspects will be discussed in a thematic manner, as set out in the sections that follow, illustrated with photographs of the key players and significant occasions, as well as other relevant images. It is to be noted that brief histories and reflections on FAOB(MB) have been previously published by the following authors: Linnane,^3^ in 1978 on the occasion of the first Congress of FAOB; Svasti,^4^ in 1981 just before the tenth anniversary of FAOB; Svasti in 1992,^5^ on the occasion of the 20th anniversary of FAOB; Anraku,^6^ in 2005 on the 50th anniversary of IUBMB and the 33rd anniversary of FAOBMB; Svasti and Sawyer,^7^ on the occasion of the joint 20th IUBMB‐11th FAOBMB Congress held in Kyoto, Japan, in 2006; de Jersey,^8^ in 2010 on the occasion of the joint 12th IUBMB‐21st FAOBMB Conference held in Melbourne, Australia, known informally as OzBio2010.

This article is not only based on these published histories, but also on the analysis of documents of FAOB(MB) Council meetings available to the authors, and certain items of correspondence and personal memories of the authors. Further significant and insightful sources of information and commentaries about FAOB(MB) are contained within the Reflections of Past Office Bearers, included as an Appendix within this History of FAOB(MB).

## 2 GOVERNANCE AND ADMINISTRATION

### 2.1 The early years of the Federation

The first small group of Office Bearers of FAOB worked hard to overcome difficulties due geographical distances within the Asia‐Oceania region and the communication barriers that were prevalent in the era before email and internet. This EC, initially a triumvirate, held correspondence amongst themselves and met occasionally at other scientific Conferences or Congresses or when the opportunity arose from international visits between them (see e.g. Figure 2). There was much correspondence and, wherever possible, personal interaction with biochemists in the Asia‐Oceania region. As the number of Constituent Members grew, in part stimulated by encouragement from FAOB Officers to form national societies of biochemistry,^3^ broader meetings of FAOB Council took place to include the delegates of Constituent Members. Thus, in 1976, at the instigation of the second President, Tony Linnane (Figure 3) there were two such meetings, the first in February 1976 at Mahidol University, Bangkok, Thailand, that extended over 3 days (although five delegates of the then 10 Constituent Member societies were unable to attend). The second meeting took place in July of that year at the 10th IUB Congress in Hamburg, Germany (where some of delegates not at the first meeting could then attend). This pair of meetings reviewed the progress of the Federation over its first three full years (1973–1975), encompassing finances of the Federation, its activities in terms of conferences, newsletter, and the number and terms of office of members of the EC.

From 1973, FAOB had sponsored a small number of symposia, three in India and one in Japan, and in 1975 plans were being made for the first FAOB Congress, as such, to take place in Japan in 1997 (see section on Congresses below). There were evident difficulties in the preparation and circulation of a regular newsletter of the Federation, the responsibility of the then Secretary General/Treasurer. To deal with such issues and to set the Federation administratively on a broader footing, decisions were made at those Council meetings in 1976 to have separate positions for Secretary General and for Treasurer, and to establish the positions of Past‐President and President‐Elect.

### 2.2 Constitutional aspects

The terms of office eventually settled down into a pattern, such that the President‐Elect served 1 year, then took up a 3‐year term, with another 2 years as Past‐President (constituting a 6‐year cycle). The cycle runs so that the incoming President‐Elect takes office and joins the EC of the Federation in the year following the completion of the term of the Immediate Past‐President. This is unlike the process in IUB(MB) whereby there is a 9‐year cycle, in which the President‐Elect serves 3 years (coincident with the 3‐year term of the Immediate Past President), before the 3‐year term as President commences.

The terms of office of Secretary General and of Treasurer were set at 3 years in the first instance; eligibility was enabled of the incumbent for election to a second (and final) term of 3 years. These terms of office of the members of the EC are all set out in the constitutional documents of FAOB(MB), initially termed the Statutes, determined by the Council (see below for the evolution of the Statutes into the documents of the present day). The positions on the EC were drawn initially from amongst the delegates of Constituent Member Societies or Groups but later became positions for which nominations could be received from any individual members of Constituent Member Societies or Groups. Elections took place within the Council (which included all the delegates plus the members of the EC).

The constitutional documentation of FAOB(MB) has evolved over the years from Foundation Statutes of 1972, which was a relatively simple three‐page document with just nine sections, through to the formal documents of today (that take up a combined 62 pages—these documents can be found on the FAOBMB webpage at: www.faobmb.com/about-faobmb/constitution/).

Currently, there are the Rules of FAOBMB (organised in the format prescribed by the State Government of Victoria, Australia, initially adopted when the Federation became an Incorporated Association in 1992; see below), the Standing Orders of FAOBMB (which prescribes and limits the day‐to‐day governance and procedures of the Federation), and the Appendices of the Standing Orders (which, in Appendix I sets out monetary sums for the Federation's various activities, and in Appendix II describes the Gender Equality Policy of FAOBMB).

In 1983, the Fellowships Committee was constituted as a sub‐Committee of Council and, likewise, the Education Committee was constituted in the mid‐1990s. At first the Chairs of these Committees were appointed from amongst the delegates. Later they were elected by members of Council in their own right, as for the Secretary General and Treasurer, and for similarly defined terms (3 years plus one possible second such term). The Committee Chairs were required to submit reports to Council and, from the 1990s, were encouraged to attend meetings of Council, but only as observers. In 2008, Council determined that these two Chairs would be voting members of Council. Holders of these two positions became formally members of EC when the major revision of the Rules and Standing Orders of FAOBMB took place in 2013. Those revisions brought the number of positions on the EC to six.

The establishment of FAOB as an Incorporated Association took place in 1992, 20 years after the foundation of the Federation. This was driven by as much by financial considerations (set out in the following section, relating to the need to get tax‐exempt status for the Federation), as much as in context of sound governance and administration. In the latter context, incorporation provided the means for the Federation becoming a recognised legal entity, with governance established via the EC and Council under a defined jurisdiction, namely the State of Victoria, Australia. The process of incorporation had commenced following discussions both within FAOB and with other people interested in the welfare of FAOB, as to how the Federation could be best organised. In August 1991, Tony Linnane (then Treasurer of IUB) discussed these matters with N. V. (Ram) Bhagavan (then Treasurer of FAOB) at the 15th IUBMB Congress in Israel. This meeting resulted in the suggestion to EC that FAOB be incorporated in Victoria, Australia. With the subsequent approval of Council, EC then liaised with Barry Preston in Melbourne. Preston was an academic staff member at Monash University, who assisted Linnane in his IUB work. On behalf of FAOB, Preston arranged for a lawyer in Melbourne, Alan McQuillan, to be given the task of drawing up the documents for incorporation, and to draw up the draft Rules of FAOB to comply with the Associations Incorporation Act 1981 in the State of Victoria. At the same time, McQuillan would also seek to get tax‐exempt status for FAOB, through the Australian Taxation Office. After much passing back and forth during 1992 of many drafts of the Statement of Purposes and Rules, under the leadership of FAOB by President Jisnuson Svasti, the appropriate Resolutions were passed by Council at its meeting in Shanghai, China, on 15 November 1992. The documentation was sent by fax to McQuillan in Melbourne, who then submitted the necessary papers to the Registrar of Incorporated Associations in the State of Victoria. The Certificate of Incorporation of FAOB was duly issued by the Registrar, dated 8 December 1992. In the meantime, following McQuillan's submissions on behalf of FAOB to the Australian Taxation Office, the Office of the Deputy Commissioner of Taxation in Melbourne wrote to McQuillan on 18 September 1992 advising him that the income of the Federation is considered to be exempt from income tax under the relevant section of Australian Law. A large amount of work to achieve these objectives was carried out by the EC at the time, working with McQuillan, who was of course paid for his services by FAOB.

As part of the process of incorporation, another officer of FAOB had to be appointed, initially called Public Officer, the first such incumbent being William H. Sawyer (who later became President of FAOBMB; Figure 6). This position became renamed as Secretary after 2012 due to changes in the relevant Victorian law (these changes also precipitated the major revision of the Rules and Standing Orders that took place under the stewardship of Secretary General Phillip Nagley in 2013–2014). This Public Officer/Secretary position can only be held by a person resident in Australia, the incumbent being the official contact of the Federation, responsible for submitting a brief annual financial statement and for notifying any changes in the Rules of the Federation (but not the Standing Orders), as may occur from time to time. Oddly, the official address of FAOBMB and that of its various bank accounts in Australia, is now the home address of the Public Officer/Secretary. The list of such Public Officers/Secretaries of FAOBMB, each of whom is (or who has been) a member of the Australian Society of Biochemistry and Molecular Biology (ASBMB), and residing in Melbourne, Victoria, can be found on the FAOBMB webpage at: www.faobmb.com/about-faobmb/history-of-faobmb/.

Shortly after incorporation of FAOB in 1992, the Federation resolved at the meeting of Council in Taipei, China, on 7 December 1993, to change its name to FAOBMB. Fortunately, the preparation of the documentation was not as onerous as for the initial incorporation, but still required extensive liaison between the outgoing Secretary General Kay Hoon Lee and his incoming counterpart Qi‐Shui Lin, with Bill Sawyer and Alan McQuillan in Melbourne. A new set of Rules for FAOBMB had to be prepared and submitted to the Registrar, together with appropriate Resolutions of Council signed by two EC members, President Ram Bhagavan and Secretary General Qi‐Shui Lin. The new Certificate of Incorporation of FAOBMB was issued by the Office of the Registrar on 30 May 1994.

### 2.3 Office bearers of the Federation

On the abovementioned webpage dealing with the history of FAOB(MB), are listed, in chronological order for each position, all the Office Bearers of FAOB(MB) from the foundation of the Federation in 1972 to the present day. It is interesting to consider the data in Table 3, in which are set out the countries represented by these Office Bearers over the 50‐year history of the Federation. Of the founding societies, Japan (14 total) and Australia (eight total) have the highest representation of Office Bearers, especially Japan as a source of seven Presidents. This reflects the strong support that FAOB(MB) has received from the Japanese Biochemical Society (JBS), especially in the early decades of the Federation. The Australian contribution to Office Bearers is more broadly spread, with the most being in the Offices of President and Secretary General (three each). The Secretary General position has been held by incumbents from English‐speaking countries for the most part, a feature that is consonant with English being the official language of the Federation. Of the other countries, China (Beijing), India, Korea, Malaysia, Singapore and Thailand have had multiple representatives. Three individuals stand out in the data assembled in Table 3, as having held three separate Offices in FAOB(MB), namely, Qi‐Shui Lin (China, Beijing), Ram Bhagavan (Hawaii) and Takashi Murachi (Japan). The contributions of these, and many other Office Bearers, who have been energetic and dedicated individuals, cannot be over‐estimated in the development of the Federation.

**TABLE 3:** Countries represented by Office Bearers of the Federation of Asian and Oceanian Biochemists (and Molecular Biologists)^a^

Office/country^b^	President^c^	Secretary General^d^	Treasurer^d^	Fellowships Chair^d^	Education Chair^d^
Australia	3 Edwin C. Webb (1972–1974) Anthony W. Linnane (1975–1977) William H. Sawyer (1999–2001)	3 Fyfe L. Bygrave (1980–1987) John de Jersey (2006–2011) Phillip Nagley (2012 – 2017)		1 Paul Gleeson (2018–current)	1 Susan Hamilton (2002–2005)
China, Beijing	2 Qi‐Shui Lin (2002–2004) Zengyi Chang (2017–2019)	1 Qi‐Shui Lin (1994–1999)		1 Qi‐Shui Lin (2007)	
China, Hong Kong			1 Shannon Au (2020–current)		
China, Taipei	1 Andrew H.J. Wang (2011–2013)				
Hawaii	1 Nadhipuram. V. Bhagavan (1993–1995)		1 Nadhipuram. V. Bhagavan (1987–1992)		1 Nadhipuram V. Bhagavan (1997–2001)^e^
India	1 Bimal K. Bacchawat (1984–1986)	1 N.R. Mougdal (1972–1975)	1 N.R. Mougdal (1972–1975)	1 Samir K. Brahmachari (2004–2006)	
Japan	7 Kazutomo Imahori (1978–1980) Osamu Hayaishi (1981–1983) Takashi Murachi (1987–1989) Yasuhiro Anraku (1996–1998) Masamitsu Futai (2008–2010) Kiyoshi Fukui (2014–2016) Akira Kikuchi (2020–2022)	1 Takashi Murachi (1976)	3 Takashi Murachi (1976) Yasuhiro Anraku (1995–1995) Kiyoshi Kita (2002–2007)	3 Takashi Murachi (1983‐1986) Masamitsu Futai (1993–1995) Yuzuru Ishimura (1999–2002)	
Korea	1 Kyung‐soo Hahm (2005–2007)		2 Si Myung Byun (1996–2001) Uhtaek Oh (2008–2013)	1 Sang‐Sup Lee (1990–1992)	
Malaysia		1 Sheila Nathan (2018–current)		2 Perumal Ramasamy (2003) Sheila Nathan (2014–2017)	
New Zealand		1 Mervyn G. Smith (1997–1999)			
Philippines					1 Gracia F.B. Yu (2018–current)
Singapore		2 Kay Hoon Lee (1998–1993) Hoon‐Eng Khoo (2000–2005)		1 Raymond Yuen (1996–1998)	2 Hoon‐Eng Khoo (2006–2011) Siok‐Im Koh (2012–2017)
Thailand	1 Jisnuson Svasti (1990–1992)		3 Serene Vimokesant (1977–1980) Jisnuson Svasti (1981–1986) Piamsook Pongsawasdi (2014–2019)	2 Montri Chulavatnatol (1987–1989) Piamsook Pongsawasdi (2008–2013)	
Total	17	10	11	12	5

^a^ The Federation changed its acronym from FAOB to FAOBMB in 1993, with the official change of its registered name.

^b^ Countries are listed in Alphabetical order where an entry is relevant. Countries are omitted if there are no representative Office Bearers (namely, Bangladesh, Indonesia, Iran, Myanmar, Nepal, Pakistan, Sri Lanka, and Vietnam).

^c^ Data from Table 1.

^d^ Data from FAOBMB webpage at: www.faobmb.com/about‐faobmb/history‐of‐faobmb/. Where Office is listed there under Secretaries‐General/Treasurers, the country of representation is given for both Offices in this Table, Secretary General and Treasurer.

^e^ It is not clear from the available records of FAOBMB as to which year the office of Education Chair commenced.

Regarding the gender distribution of Office Bearers, whose names are listed in Table 3, Presidents have been exclusively male over the 50‐year history of FAOB(MB). Three of the other Offices (Secretary General, Treasurer, Fellowships Chair) have had about 80% male and 20% female occupancy over these years. On the other hand, the Education Chair has been overwhelmingly a province of females (80% female, 20% male). In light of the Statement on Gender Equality produced by FAOBMB in 2015 (available on the FAOBMB website at: www.faobmb.com/about-faobmb/constitution/), these proportions are not generally consonant with such aspirations but they do reflect the nature of the scientific communities and the leadership over the decades from the 1970s to the present. It is heartening, however, to consider the changes in the proportion of females amongst Office Bearers of FAOB(MB) over the five decades of the history of the Federation (Table 4). The most recent decade has seen a huge jump in the proportion of females among the Office Bearers of FAOBMB, such that the desired equality of proportion in respect of gender has finally been achieved. FAOBMB works hard, together with the Organisers of Congresses and Conferences to improve the proportion of females among both the Organising Committees and the invited speakers to these events sponsored by FAOBMB.

**TABLE 4:** Gender distribution, by decade, in office bearers of the Federation of Asian and Oceanian Biochemists (and Molecular Biologists)^a^

Decade^b^/Gender^c^	1972–1982	1983–1992	1993–2002	2003–2012	2013–2022
Male	10	8	12	10	4
Female	1	0	2	2	5
Total	11	10	14	12	9
Percentage male	91	100	86	83	44
Percentage female	9	0	14	17	56

^a^ The Federation changed its acronym from FAOB to FAOBMB in 1993, with the official change of its registered name.

^b^ Entries for each decade are made based on the first year of Office held by an individual being within the date range indicated.

^c^ Data concerning Office Bearers are from Table 3. Gender information, although not shown in Table 3, was recognised in those lists by J. Svasti and P. Nagley. Data are included neither for Archivist nor for Public Officer/Secretary positions.

### 2.4 Logo of the federation

**FIGURE 8:**
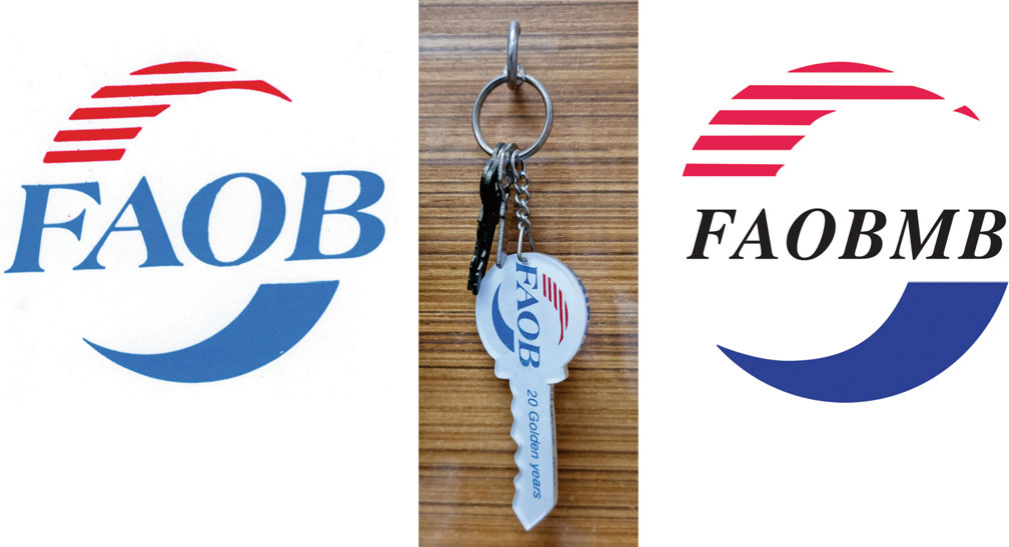
Logos of FAOB(MB). Left panel: FAOB logo adopted by Council in 1989. Centre panel: Souvenir Keyring distributed in 1992 for the 20th Anniversary of FAOB. Right panel: new FAOBMB logo generated after the change of name of the Federation in 1993. Image of keyring provided by J. Svasti

The logo of the Federation has an interesting history. At first, a logo in the form of a stylised weathercock was provided by Kunio Yagi (Japan) for use by FAOB on official documents, letterheads for correspondence, and on newsletters. This logo can be seen in the example of the FAOB Newsletter from 1987 (see below). But that logo turned out to have remained the property of Yagi himself and in the late 1980s he indicated that he wished to use the logo as part his own developing scientific and technical organisations. Therefore, FAOB had to develop a new logo of its own. Under the stewardship of Secretary General Kay Hoon Lee, a competition was held in 1989 for delegates to submit a design for a new logo, with a prize of $200 for the winning entry. Accordingly, at the Council meeting held in Seoul, Korea in August 1989, voting took place on 11 logo designs that had been submitted for consideration. The winning logo was that submitted by the Korean delegate, Sang‐Sup Lee, who was also from the Organising Committee of the 5th FAOB Congress held in Seoul that year (the winning design having been modified from the logo of that Congress). This logo can be seen in Figure 8, in the left panel. The official description of the logo, in somewhat lofty language, reads as follows on the occasion of its official adoption:

‘The upper part represents the helical structure of DNA which is the basic unit of life. Thus, it indicates that the Federation is in activities relating to those of life sciences. The circular nature of the logo reflects the continual flow of information in the field of biochemical sciences without interruption. It also reflects the dynamism of the Federation. The broken parts (stripes) of the logo are also to represent members from Asian Countries and the solid part members from Oceanian Countries or vice versa’.

This FAOB logo was incorporated into the design of a souvenir keyring distributed among the FAOB community on the occasion of the 20th Anniversary of the Federation in 1992 (Figure 8, centre panel). The particular keyring photographed is still in use and hangs in an office at Mahidol University, Bangkok. But by the following year, the Federation had changed its name to FAOBMB, and the logo had to be redesigned accordingly (Figure 8, right panel). An initial redrawing of the logo had the lettering of FAOBMB protruding at each edge of the logo, examples of which can be seen in the FAOBMB Bulletin of 1994, or the cover of the first issue of the FAOBMB Journal in 1997 (see below). A later version of the FAOBMB logo, dating from about 2010, made the logo more compact as in Figure 8. Note that the lettering in the logo for the acronym FAOB of the Federation was originally blue but became black for FAOBMB. Perhaps this was so the colour of the acronym would not to be aligned with either the blue or the red that represented the broad geographical regions covering the Federation, namely, Asia and Oceania!

### 2.5 Purposes of the Federation

Before dealing with the functions and activities of FAOB(MB) it is useful to look at the development of the Statements of Purpose of the Federation, as have appeared in the constitutional documents over its history. In the Foundation Statutes of 1972, the Objects of the Federation were stated to be as follows.

‘The Federation exists to promote the science of biochemistry, in particular by the dissemination of information, by arranging meetings and in other ways encouraging contacts between its members. In the furtherance of these aims, the Federation also intends to work closely with other bodies having similar objectives to the Federation, and with the International Union of Biochemistry’.

This exact wording remained the same until the establishment of FAOB as an Incorporated Association in 1992, when the Statement of Purpose was required to be set out as numbered clauses, to comply with the relevant Act of Parliament of the State of Victoria (at that time, the Associations Incorporation Act 1981). Today, the wording of the Purposes is as follows (under the Associations Reform Act 2012, the Rules now being taken as the terms of a contract between the Association and its members):

1. To promote the science of biochemistry and molecular biology, including education, research and applications, worldwide and in particular in the Asian and Oceanian Region.

2. To disseminate biochemical and molecular biology information among its members and others.

3. To arrange and conduct meetings, conferences, workshops and symposia to increase and promote the knowledge of biochemistry and molecular biology among its members and others.

4. To encourage and assist the exchange of biochemical and molecular biology information among its members and others.

5. To maintain and promote close relationships with any other organization having similar purposes to the Federation especially the International Union of Biochemistry and Molecular Biology.

6. To manage the funds and other assets and liabilities of the Federation for the object of carrying out the aforesaid purposes and not otherwise.

7. To give consideration to gender and regional equity in the conduct of its activities.

The Purposes of the Federation, therefore, have remained relatively stable over the past 50 years, although there are some notable differences between the original wording and that used today. Molecular Biology was added to Biochemistry, at each mention, after the change of name of the Federation in 1993. The scope of the ‘science of biochemistry’ has been specified to include ‘education, research and applications’, and the target area for promotion of such science is now global with emphasis on the Asian and Oceanian Region. The dissemination, promotion and exchange of information is likewise targeted more broadly than just to the members and includes others (who may be scientists in other disciplines or be members of the general community, including school or university students). The nature of the meetings is better defined to include particular activities (e.g. conferences and workshops). IUBMB is referred to instead of IUB. The management and use of the funds of the Federation is restricted to the furtherance of the purposes (and not otherwise). Gender and regional equity is now specifically mentioned.

It is worth noting that the mention of gender and regional equity first appeared in the wording of the Purposes in 2014, although a specific policy statement on this aspect was not finalised until 2015 (Appendix II of the Standing Orders, mentioned above). It is also interesting that although FAOBMB had established an Educational Committee in the mid‐1990s it was not until 2014 that the word ‘education’ appeared in the first clause of the Purposes. This shows that, as with other similar organisations, the Federation moved to institute new policies and activities well before they became enshrined formally in the constitutional documents.

### 2.6 Membership categories of the Federation

An interesting consequence of the incorporation of FAOB in 1992 was that the membership of the newly formed Incorporated Association was defined as being comprised of organised bodies (namely the national societies or groups), rather than individuals. In contrast, the original Statutes of FAOB dating from 1992 stated that ‘All members of the constituent societies are members *ipso facto* of the Federation, with equal privileges’. This wording remained in the Statutes until 1991. The then newly written Rules of FAOB in 1992 enshrined two categories of membership: constituent membership and special membership. In 1994, the Rules of FAOBMB were expanded to allow a further class of membership denoted honorary membership.

The significance of these membership categories can be briefly considered. Individuals cannot join FAOBMB (this is often a source of confusion but is clearly set out on the FAOBMB website at: www.faobmb.com/about-faobmb/regional-role/). That said, members of EC are individuals, as are the delegates of Constituent Members; together these two groups of individuals make up the members of FAOBMB Council.

Special Members refer to companies that may sponsor the activities of the Federation, which are discussed later in the section on Financial Aspects. The third category, honorary membership introduced in 1993, represents an honour bestowed by Council on organisations and individuals who have made significant contributions to the Federation. The first two Honorary Members were Keizo Saji (President of the Suntory Ltd, a company in Japan, whose contribution to FAOB will be mentioned in more detail in the section on Congresses, below) and Kunio Yagi (Figure 4). Yagi had been one of the Japanese scientists who encouraged the formation of FAOB before its inception and made lifelong contributions to the development of the Federation, although Yagi was never an Office Bearer of FAOB. Among Yagi's many contributions was his provision of the first logo of FAOB, the organisation of the first FAOB Congress in 1977 in Nagoya, Japan, and the generous endowment he made in 1993 to FAOB to establish the Kunio Yagi lectureship that provides a Plenary lecture at each FAOBMB Congress.

In later years, honorary membership has been bestowed on many Past Presidents of FAOB(MB). A full list of Honorary Members can be found on the FAOBMB website at: www.faobmb.com/about-faobmb/honorary-members/.

## 3 FINANCIAL ASPECTS

### 3.1 General aspect concerning income

The income to FAOB(MB) over the years has been drawn from membership subscription fees plus other funding from IUB(MB) and other organisations (and individuals) for specific purposes. The budgetary information for FAOB(MB) has always been expressed in United States dollars, except for the Annual Statements submitted to the State of Victoria, where the monetary sums are expressed in Australian Dollars. All amounts mentioned below refer to United States Dollars. A general principle, which has never been changed since foundation of FAOB, is that irrespective of the rate of subscription paid by a Constituent Member, there is one delegate (or an alternate) appointed by the national society or group to be a member of Council.

### 3.2 Subscription fees for Constituent Members

At the outset, subscription fees for Constituent Members were established to take into account the different sizes of the various national biochemical societies. On foundation in 1972, the FAOB membership rates were set at $0.25 per member of the national biochemical society, with a minimum fee of $10 for any Constituent Member. In these early years of FAOB there were great disparities in the fees paid. The largest societies were those from Japan, Australia and India, each paying hundreds of dollars each year (in Japan's case more than $1,000). As of 1975, the remainder of the 10 Constituent Members at the time each paid <$30, with two on the base rate of $10. The financial situation for FAOB remained very tight for two further decades, and gradually improved as some of the other national societies greatly increased their membership numbers (such as Korea) and other societies with relatively large numbers of members later joined the Federation (such as China, Taipei and China, Beijing). Unfortunately, in the early years to 1975, FAOB had already encountered issues in receiving regular financial subscriptions from the smaller Constituent Members, for which the national biochemical societies in developing countries of Asia may have had only a handful of members, together with problems of currency transfer internationally. Such issues of delayed payment of subscription fees continue to the present day.

In 1981, at its Council meeting in Bali, Indonesia, FAOB introduced a stratified algorithm for determining the subscription fees to provide a more beneficial outcome based on the perceived abilities of Constituent Members to pay. This algorithm had three categories or tiers, into which each Constituent member would self‐nominate, subject to approval by Council. The initial aim was to get a subscription fee for each Constituent Member close to 10% of the income of that national society or group. In 1981, the tiers were first set at rates of $0.25, $0.50, $1.00 per member, with a minimum subscription of $20. This fee structure was retained in this form until 1994, when the addition of a fourth tier (in the middle of the range) took place. In that same year an approximate 40% increase in rates overall was made, with the minimum subscription rising to $30. The four‐tier system remained in place for 25 more years until 2019, with moderate increases in the subscription rates occurring in 2002 (25% increase; minimum fee $100) and 2010 (approximate 40% further increase; minimum fee $140).

After 2010, the disparity in subscription rates under this system began to grow, such that Constituent Members with the highest subscription fees (particularly JBS) seemed to be paying more than their fair share, and self‐nomination to a particular tier became a matter with the potential for destabilisation. Accordingly, in 2019, under the stewardship of Treasurer Piamsook Pongsawasdi, a new scheme was introduced to take effect from 2020. In this new system, detailed in Appendix I of Standing Orders (www.faobmb.com/about-faobmb/constitution/), there are three Groups (representing income levels of countries defined according to the official World Bank List of Economies) plus five Subgroups representing the number of members in the national society or group (those shown in Table 1). Each combination of Group/Subgroup has a Unit Multiplier associated with it; the value of the Unit expressed in United States dollars is fixed by Council from time to time. At its inception, the Unit Multipliers and the Unit value were chosen to provide minimal overall disruption to the fees levied under the previous system. The new system is considered to generate a more objective assignment of Constituent Members to a particular level of subscription fees, and to provide a means of dealing in the future with changes in economic situations faced by individual countries and large fluctuations in the number of members in national societies or groups (as well as an easy means to adjust fees overall due to inflation).

### 3.3 Special Members

In 1985, steps were commenced to embark on a new program of ‘special membership’ of FAOB, aimed at fund‐raising primarily from commercial organisations active in the technical areas related to biochemistry. The Statutes were amended in 1989 to include the new category of Special Member, as ‘organisations which have an interest in the objects of the Federation’. A Special Member would not be represented on Council as a voting member but could send a representative to attend Council meetings as an observer. The Special Member fee was set at annual subscription of $1,000.

The first two Special Members were admitted in 1991, by invitation of EC, namely, Millipore Intertech Asia (based in Hong Kong) and Boehringer‐Mannheim (Far East) P.L. (based in Singapore). These two companies were honoured at the 9th FAOB Symposium held in Hong Kong in December 1991, with the presentation to the representative of each company with a commemorative plaque. The number of Special Members grew rapidly over the next few years to a peak of about 20 in the mid‐1990s, mostly involving companies in Japan, plus other international companies based in Hong Kong or Singapore. By 2000, there had been some turnover in the Special Members with several companies from Korea joining this group, while others had dropped off the list. Despite many invitations from EC to other companies this list did not grow. Indeed, it seems that after 2000, when the Council documents for that year recorded a list of 19 Special Members, there were very few subscriptions actually being paid (three were paid in 2001 and none thereafter). The category of Special Members has remained within the Rules of FAOBMB with the same annual subscription fee of $1,000, but there has been no occupancy of that category for more than 20 years. Nonetheless, the funds raised over the years from Special Members, and from donations from other companies and Institutions on a ‘one‐off’ basis during the decade from 1991 to 2000, did provide a substantial boost to the coffers of FAOB(MB).

### 3.4 Support from IUB(MB)

IUB was a reliable and nurturing partner for FAOB from the outset. At first, IUB provided funds to specific events organised under the auspices of FAOB, such as Congresses and Symposia. Eventually, when the partnership between IUBMB and FAOBMB became fully established, the Treasurer of FAOBMB would provide a provisional budget covering the next triennium, to fit in with the three‐year forward budgeting process of IUBMB. Within this provisional budget of FAOBMB there eventually was a sum of $15,000 to support one triennial Congress, and $10,000 for each of the Symposia or Conferences of FAOBMB in the intervening years. IUBMB in recent years has dispensed with this arrangement and now provides $15,000 each year for the Congress or Conference of FAOBMB, without the need for a formal submission from FAOBMB, although a report is required on the activity for which the funds have been expended. IUBMB also provides funds for other activities of FAOBMB, including Young Scientist Program (YSP) events, which are held in conjunction with Congresses and Conferences jointly held or co‐labelled with IUBMB (see sections on Congresses, and on Fellowships, below). In addition, IUBMB supports an Education Symposium or Workshop now held at each of the annual scientific meetings of FAOBMB (see section on Education, below).

### 3.5 Other sources of income

Constituent Members of FAOB(MB) have been generous in their support of the Federation, where funds have been available and specific activities have been earmarked for support. Thus, as discussed below under the relevant sections, such support has been provided, for example, for Plenary Lectures at Congresses, Symposia and Conferences, for Travel Fellowships to such scientific meetings of FAOBMB, and for fellowships for YSP events.

Individuals have also been generous in support of the Federation, in respect of providing endowed funds for Plenary Lectures and for the Young Scientist Awards held at Congresses of the Federation (see relevant sections below on Congresses, and on Awards, respectively, below). A major source of funding for the other Awards of FAOBMB has come from foundations in Taipei, China, organised by Andrew Wang, President from 2011 to 2013 (Figure 7), as discussed under the section on Awards, below.

### 3.6 Location of bank accounts and tax‐exempt status of the federation

The bank accounts in which funds of the Federation have been held usually have comprised a Working Account at a location convenient for the Treasurer. This has sometimes been a local account held specifically in the name of the Federation, or for some Treasurers it has been a subsidiary account held in the name of the Constituent Member society or group, available for the use of the Federation. The placement of some of the funds of the Federation in a fixed location was eventually arranged when accounts were set up in Australia, at the National Bank, in Melbourne, which took place in 1992 around the time of establishment of FAOB as an Incorporated Association (see above). Before that efforts were made from time to time to set up accounts in the United States or the United Kingdom, but these offshore accounts did not last; the funds eventually were all kept within countries of the FAOBMB region.

Another major issue that the Federation had to deal with in its first two decades, in addition to its not having a fixed home address, was not having a clear statement of its status as a tax‐exempt organisation. This was remedied in 1992 as mentioned above. Before that time, this was of little consequence for the day‐to‐day operations of the Federation in dealing both with the Constituent Members and IUB(MB) but it was a potential problem for some companies and individuals who wished to donate funds to the Federation to support its activities. The implications of this for support provided to at least two of the Plenary Lectures at FAOBMB Congresses are mentioned below.

## 4 CONGRESSES, SYMPOSIA AND CONFERENCES

### 4.1 The cycle of annual scientific meetings

The first FAOB Congress took place in October 1977, in Nagoya, Japan.^3^ This was 5 years after the foundation of FAOB, with the Federation having earlier contributed to the sponsorship of a few other symposia in the region but never having organised one under its own banner, as such. This was a great step forward, despite the financial risks that prevailed at the time due to the small amount of funds at the disposal of the Federation. The Congress included two symposia sessions of interest to biochemists in the region: ‘Biochemical Aspects of Nutrition’, and ‘The Structure and Function of Biomembranes’ with other oral and poster sessions on various topics. The Congress attracted about 500 scientists, including visiting speakers from Europe and the United States, representatives of all 14 Constituent Members at the time from the FAOB region (Table 1), together with members of the IUB EC and representatives from the two other regional Federation then associated with IUB, namely, FEBS and the Pan‐American Association of Biochemical Societies (the forerunner of PABMB). This grand launching of FAOB in the international biochemical arena, a strategy planned by FAOB President Tony Linnane and his colleague the local organiser Kunio Yagi, was generously supported by the JBS.^3^

The Federation had envisaged a series of triennial Congresses for the future, with smaller Symposia in the intervening years, all at different locations in the region. Under this plan, there would be an annual scientific gathering of biochemists within the FAOB region, at which the annual meeting of Council would also take place. This plan was effected such that the first and second FAOB Symposia took place in Singapore in 1978, and Kuala Lumpur, Malaysia in 1979, respectively. The second FAOB Congress was held in Bangalore, India in 1980. Following the third FAOB Symposium in Bali, Indonesia, in 1981 the cycle was broken by the 12th IUB Congress that was held in Perth, Australia in 1982 (when no scientific meeting of FAOB took place). Nonetheless, the annual Council meeting took place in Perth that year, during the IUB Congress.

The cycle of FAOB Congresses and Symposia then resumed in 1983, with the third FAOB Congress in Bangkok, Thailand, and in 1984 the fourth FAOB Symposium in Manila, Philippines. These brief mentions illustrate the way in which these annual scientific meetings moved around the Asia‐Oceania region, being enthusiastically hosted by the local national biochemical society or group, supported by limited funding from FAOB enabling the travel and accommodation expenses of a small number of high‐profile international speakers at each such scientific meeting. The benefits of such arrangements to the local biochemistry community in the host countries were, therefore, considerable in terms of contact with FAOB delegates and other visiting scientists and students from the regions, as well as being able to hear much about the latest research and methodology from the visiting international speakers. These meetings also allowed the host countries to showcase their own research activities, as the vast majority of participants in these FAOB Congresses and Symposia were from the host country itself. The scientific programs were organised to enable many of the local scientists to present their work in colloquia and poster sessions.

The full list of these annual scientific meetings of FAOB(MB) can be found on the FAOBMB webpage at: www.faobmb.com/news-and-events/past-congresses-conferences-and-symposia/. Note that after 2009, the term ‘Symposium’ for the scientific meetings between Congresses was replaced by ‘Conference’. This was because confusion was developing as to the term ‘Symposium’: it was being used not only for the full scientific meeting every second or third year of the annual cycle but also for focussed 2‐h sessions within a full scientific meeting. The expression ‘Colloquium’ was then falling out of use for such smaller oral sessions. In addition, scientific meetings in the region had become more complex than in the early days of FAOB, with sessions involving symposia on different topics being scheduled in streams that ran parallel to each other, necessitating individuals to choose which of the simultaneous sessions to attend. Plenary lectures were held, of course, delivered by the major speakers, with nothing else scheduled at those times.

Guidelines for the Organisation of Congresses and Symposia were first prepared in 1991, for the information of prospective and engaged Local Organising Committees, as well for EC and Council. The details in these Guidelines eventually found their way into the Standing Orders of FAOBMB. The Guidelines are updated from time to time to keep all concerned informed of the current policies and practices of the Federation.

### 4.2 Disruptions to the cycle of scientific meetings

The cycle of triennial Congresses and Symposia/Conferences set up in 1977 has continued in broad terms to the present day. There has been a small number of interruptions to the schedule where scientific meetings have been cancelled or relocated at the last minute, due to unforeseen circumstances, notably the following. The 8th FAOB Symposium scheduled for Dhaka, Bangladesh in December 1990 did not take place due to political unrest in the host country. No replacement scientific meeting was organised at the same scale to replace the cancelled scientific meeting but a smaller Symposium was later reconvened in June 1991. In the meantime, the postponed Council meeting from 1990 was held on 25 March 1991 in Bangkok, Thailand (where the President Jisnuson Svasti was based). A second Council meeting took place later in 1991 on 15 December, at the 9th FAOB Symposium in Hong Kong.

Shortly after the tragic events of 11 September 2001 in New York and other parts of the United States, the disruption of the international travel plans of many scientists led to the cancellation of the 9th FAOBMB Congress to have been held in Lahore, Pakistan, in November of that year. The Council meeting that year was rescheduled to Singapore on 11 November 2001. While no replacement FAOBMB Congress was held, 4 years later the 18th FAOBMB Symposium was successfully held in Lahore, Pakistan during November 2005. Interestingly, following cancellation of the 2001 FAOBMB Congress, the cycle of FAOBMB scientific meetings was adjusted by 1 year such that the 10th FAOBMB Congress was held in Bangalore, India, in 2003. This brought the cycle of FAOBMB Congresses exactly in phase with that of the IUBMB Congresses, enabling the jointly held IUBMB‐FAOBMB Congress to be held in Kyoto, Japan in 2006 and for several such joint Congresses since then (see below for comments on joint scientific meetings).

FAOBMB scheduled another scientific meeting in Dhaka, Bangladesh, in December 2013. As had happened 23 years previously with the 8th FAOB Symposium, the 23rd FAOBMB Conference was cancelled due to political unrest in that country. Rather than rescheduling the meeting in Bangladesh as before, a substitute (but smaller) scientific meeting was organised in Singapore, covering the originally planned dates. This was termed a Special FAOBMB Symposium, and the Council meeting for 2013 was also held in Singapore on that occasion.

### 4.3 Jointly held scientific meetings

In general, the FAOB(MB) scientific meeting in any 1 year was held jointly with the annual scientific meeting of the national biochemical society or group hosting the FAOB(MB) events that year. On occasions, FAOBMB has held Congresses or Conferences jointly with larger groups, most often IUBMB. The first such jointly held Congress was the 20th IUBMB‐11th FAOBMB Congress held in Kyoto, Japan in June 2006. This was a relatively large meeting with more than 9000 registrants, 1000 international speakers and 2000 international participants. The huge numbers of local participants (about 7000) attested to the strength of the Japanese biochemical community. The Congress program was held entirely in English, as required by both IUBMB and FAOBMB.

Unusually for the IUBMB cycle of triennial Congresses, the subsequent IUBMB Congress in 2009 was also held in Asia as the 21st IUBMB‐12th FAOBMB Congress in Shanghai, China. This was also a very successful event but with participants not as numerous (totalling about 2800) as for that held in Kyoto 3 years before. The next IUBMB Congress to come to Asia was the 24th IUBMB‐15th FAOBMB Congress held in Seoul, Korea in 2018 (attracting about 3500 registrants). The next such joint Congress will be the 26th IUBMB‐17th FAOBMB Congress to be held in Melbourne, Australia, in 2024.

IUBMB initiated a new component of its cycle of scientific meetings in 1992, namely IUBMB Conferences held in each of the 2 years between Congresses (this cycle lasted until 2016, when the IUBMB Conferences were replaced with the IUBMB Focused Meetings). Interestingly, the first IUBMB Conference was held in Nagoya, Japan (when Kunio Yagi was President‐Elect of IUBMB), on the topic of ‘Biochemistry of Diseases’. Because there was an FAOB Congress that year in Shanghai, China (at which the 20th Anniversary of FAOB was celebrated; see below), the first IUBMB Conference was not held jointly with FAOB. There were, however, two FAOBMB Conferences later held jointly with IUBMB Conferences. These were the 12th IUBMB‐21st FAOBMB Conference held in Melbourne, Australia, in 2010 and the 15th IUBMB‐24th FAOBMB Conference held in Taipei, China in 2014. More recently, the 27th FAOBMB Conference was held in Kuala Lumpur, Malaysia, with special funding from IUBMB on the occasion of the Malaysian Society for Biochemistry and Molecular Biology having become a full Adhering Body of IUBMB.

One other joint meeting, of especial note in this context, was the 17th FAOBMB Symposium, held in Bangkok, Thailand in November 2004. This was co‐labelled as the second IUBMB Special Meeting and the 7th A‐IMBN Conference. The Asia‐Pacific International Molecular Biology Network (A‐IMBN) was an organisation founded in 1997 to promote the development of molecular biology and biotechnology throughout the Asia‐Pacific region. It was envisaged by its founders to become an international body of the format and stature of EMBO, but in the same region of the world as that broadly represented by FAOBMB. In respect of the parallel aims, and the identity of the regional scope, of A‐IMBN and FAOBMB it was natural for such a joint meeting to be held. The generous funding by both IUBMB (see below) and A‐IMBN made for a very successful joint conference on the general theme of ‘Genomics and Health in the 21st Century’.

A‐IMBN Conferences were held annually from 1998 to 2016, the meeting in 2004 being the only one shared with FAOBMB. The membership structure of A‐IMBN consisted of leading scientists in the region, who generally were invited to join the organisation. Funding was obtained from institutions and companies in the region, and the A‐IMBN funds were expended not only on conferences but also on training courses and workshops. A‐IMBN reached its zenith of activities around 2010, involving an electronic network of participating scientists and institutions, and a planned electronic journal of molecular biology and biotechnology; but A‐IMBN activities seemed to have declined thereafter, as revealed on the A‐IMBN website at www.a-imbn.org.

For its part, IUBMB provided strong support to the 17th FAOBMB Symposium in Bangkok in 2004. This scientific meeting was designated as an IUBMB Special Meeting that focussed on an area of interest relevant to the region in which it was held. Since IUBMB President Mary Osborn was a recipient of the UNESCO‐L'Oréal for Women in Science Award, an event was also held with the For Women in Science Thailand program. This emphasised the interest of FAOBMB in the gender issue, which became more prominent later. Other IUBMB EC members also attended that conference in Bangkok. Indeed, at many of the joint conferences held between IUBMB and FAOBMB, IUBMB EC members would attend and often there would be a formal IUBMB EC meeting. At the joint Congresses IUBMB would, of course, hold its EC meeting as well as its triennial General Assembly. The connections between IUB(MB) and FAOB(MB) over many years had been such that individual members of IUB(MB) EC would sometimes attend the Council meetings of FAOB(MB) and address the Council on significant issues in international biochemistry (and molecular biology). Further, the President of FAOBMB or a representative, is now invited to attend all meetings of IUBMB EC (as are the Presidents of the other three regional Federations).

### 4.4 Additional funding for lectures at scientific meetings

FAOB(MB) has been very fortunate to have received generous funding from several sources in support of lectures by distinguished invited speakers at Congresses, most of which are delivered in plenary sessions. Such funding has enhanced the quality of these scientific meetings, enabling international biochemists and molecular biologists of the highest experience and achievement to visit the region and present their work to large gatherings of scientists here.

As early as 1983, FAOB President Osamu Hayaishi negotiated the establishment of an endowment fund in the name of the Japanese Society for the Promotion of Science (JSPS), to which the Suntory Company had donated approximately $20,000. In turn, JSPS donated this sum to FAOB to establish the JSPS Endowment Fund, the interest from which would be used to support a plenary lecture at each triennial FAOB Congress, to be called the JBS Lecture. This seemingly convoluted arrangement was put in place to deal with the lack of explicit tax‐exempt status of FAOB, which was only resolved much later in 1992 (see section on Financial Aspects, above). The first such JBS Lecture was delivered by Hayaishi himself at the 3rd FAOB Congress in Bangkok, Thailand in 1983. He has been followed in this prestigious lectureship at FAOB(MB) Congresses by many other distinguished speakers from all over the world.

In 1993, following the establishment of separate endowments for other plenary lectures (as discussed below), the JBS Lecture was renamed the Osamu Hayaishi Lecture, as a mark of respect for his establishment of the JBS Lecture and his important contribution to FAOB during his presidency 1981–1983. To further mark the seminal role played by the establishment of the JBS Lecture, the President of the Suntory Company (Keizo Saji) was made the first Honorary Member of FAOBMB in 1993. The funding mechanism for the plenary lecture provided by this first endowment (i.e. payment of the triennial expense from interest, not capital) provided a model for the subsequent three endowments that resulted in further Named Plenary Lectures at Congresses (those of Murachi, Yagi and Svasti, as discussed below), plus a fourth endowment for the establishment of the Young Scientist Awards by Anraku (see section on Awards, below).

Following the sudden passing in May 1990 of Takashi Murachi, who was then Past‐President (having served as President 1987–1989), and who had been involved with FAOB from the time of its foundation (Table 3), there was understandable outpouring of grief in the FAOBMB community and expressions of sympathy for his family. To mark the contributions of Murachi to FAOB, under the leadership of the President Jisnuson Svasti, FAOB undertook to establish a Murachi Memorial Endowment. Through discussions with JBS and the bereaved family, Mrs Etsuko Murachi agreed to donate $20,000 to FAOB, to establish the Murachi Memorial Fund. At the 6th FAOB Congress held in Shanghai, China in November 1992, a plaque was presented to Mrs Murachi by Jisnuson Svasti on behalf of FAOB. Many personal donations, and those from institutions and national biochemical societies, enabled the fund to grow to more than $37,500 by December 1993. The interest from these funds was used to establish the Takashi Murachi Memorial Lecture to be delivered as a plenary lecture at FAOBMB Congresses.

Kunio Yagi had already indicated in 1989 that he wished to provide a similar endowment to FAOB to establish a plenary lecture. It took some years until the administrative and financial structures of FAOB (including tax‐exemption) had evolved to enable Yagi to provide the sum of $20,000 in 1993 from the Institute of Applied Biochemistry in Gifu, Japan, of which he was the founding Director. This established the Kunio Yagi Lecture at Congresses of FAOB(MB).

Meanwhile, in the same year 1993, Jisnuson Svasti provided a similar donation of $20,000 to FAOB to establish a Plenary Lecture in his name at Congresses of FAOB(MB). In this case, the terms of the endowment specified that the distinguished scientist delivering the Jisnuson Svasti Lecture must be from the FAOB region.

The four named plenary lectures mentioned above were supplemented by FAOBMB itself in 2006, when the FAOBMB Lecture was established. Funds were set aside by FAOBMB for this fifth Plenary Lecture at Congresses, commencing with the joint 20th IUBMB‐11th FAOBMB Congress held in Kyoto, Japan.

Constituent Member national biochemical societies have also provided funds for plenary lectures at FAOBMB Congresses. ASBMB provided funds for a Plenary Lecture at the 7th FAOBMB Congress in Sydney, Australia in 1995, and at the subsequent eighth Congress in Kuala Lumpur, Malaysia in 1998. Although funds were likewise promised for the ASBMB Lecture at the ninth Congress to be held Lahore, Pakistan in 2001, this Congress did not eventuate (see above). There was no further funding for a Plenary Lecture from ASBMB thereafter.

In 1999, the Society of Biological Chemists (India) (SBC(I)) reached an agreement with FAOBMB to provide funds for a Plenary Lecture termed the GN Ramachandran Lecture, named after the eminent Indian scientist who had made many contributions to biochemistry, from his background in physics. Best known to biochemists for his work that led to his creation of the Ramachandran plot for understanding peptide structure, he was the first to propose a triple‐helical model for the structure of collagen. The agreement specified that the lecture would be delivered by a scientist proposed by SBC(I) and approved by FAOBMB EC; in each case the lecturer has been from India. The first two GN Ramachandran Lectures were delivered at the 15th FAOBMB Symposium in Beijing, China in 2000 and, after the gap in the cycle in 2001, at the 16th FAOBMB Symposium in Taipei, China in 2002. Thereafter, the Ramachandran Lecture took place at Congresses of FAOBMB. In recent times, after the program of Congresses had become very congested, the agreement has been re‐negotiated such that the GN Ramachandran Lecture is now given at each Congress in a special FAOBMB Session together with the lectures delivered by the two winners of the Young Scientist Award.

At many Congresses and Conferences of FAOBMB, funds have been successfully obtained from both IUBMB and FEBS for specific eminent scientists to be sponsored by these organisations. In the case of the IUBMB Lecture, the Organising Committee and FAOBMB jointly propose to IUBMB the name of a distinguished scientist. In the case of the FEBS Lecture, support is sought from FEBS for a particular event and the relevant Officers of FEBS then propose a panel of names of European scientists for consideration by the Organising Committee and FAOBMB EC.

### 4.5 The indispensable role of congresses and conferences for the Federation

The scientific meetings of FAOB(MB) have provided the lifeblood of the Federation. They have enabled biochemists and molecular biologists in the Asia‐Oceania region to assemble and to hear the latest research and technology from the best in the world, as well as to showcase the research from the region. The rotation of the venue of for these scientific meetings around the region has brought the science directly to the scientists of the host country, particularly the younger scientists. The Travel Fellowships scheme (see next Section) has enabled younger scientists to travel for other countries within the region to participate in the Congresses, Symposia and Conferences of FAOB(MB). These scientific meetings provide an opportunity for the Council to meet in person at various locations in the region, to participate in the administrative and strategic decision‐making of the Federation, to enjoy the science itself and to partake of the local culture of the host country. Examples of the gatherings include the following: EC and Council members assembled at the 5th FAOB Symposium held in Honolulu, Hawaii in 1985 (Figure 5); and an assembly of Presidents at the 17th FAOBMB Symposium in Bangkok, Thailand in November 2004. (Figure 6).

**FIGURE 9:**
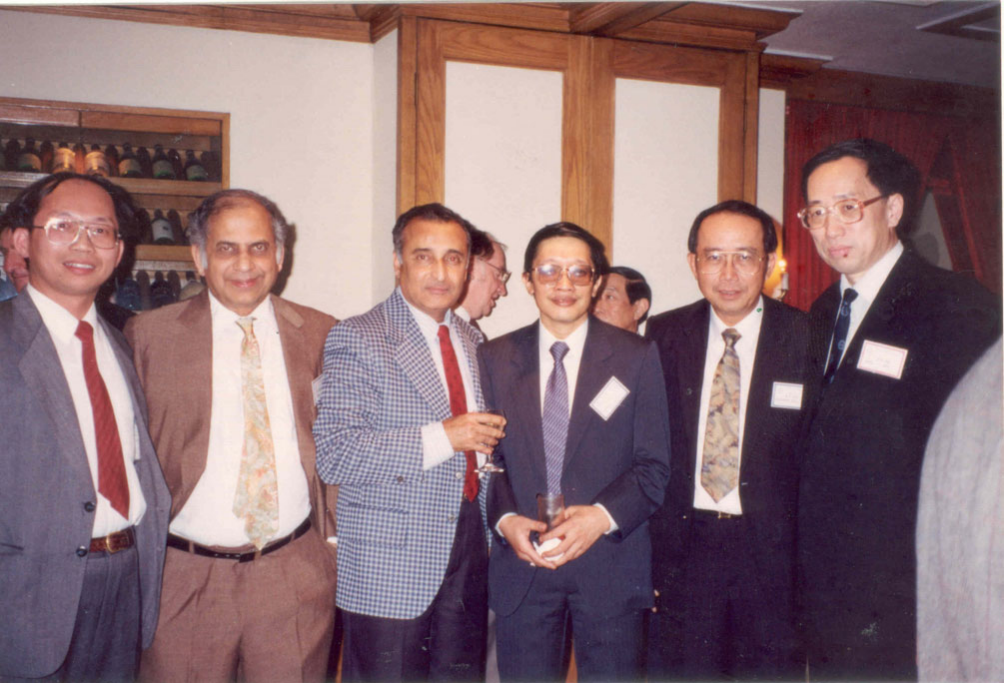
FAOB colleagues at Reception held in Shanghai, China, on 19 November 1992 to mark the 20th Anniversary of FAOB. From left: Yau‐Huei Wei (Taipei, China), Pushpa M. Bhargava (India), Nadhipuram V. Bhagavan (Hawaii), William J. O'Sullivan (Australia—*partially obscured*), Jisnuson Svasti (Thailand), Kunio Yagi (Japan—*partially obscured*), Kay Hoon Lee (Singapore), Qi‐Shui Lin (China). Image provided by J. Svasti

These scientific meetings have been the occasion of significant anniversaries, notably the 20th Anniversary of FAOB celebrated in 1992 at the 6th FAOB Congress in Shanghai, China, in November 1992. President Jisnuson Svasti went to great efforts to organise a reception to celebrate that occasion (Figure 9). The keyring memento distributed on that occasion is depicted in Figure 8. More than 40 signatures of people present at the reception were collected into a special album (not depicted here).

**FIGURE 10:**
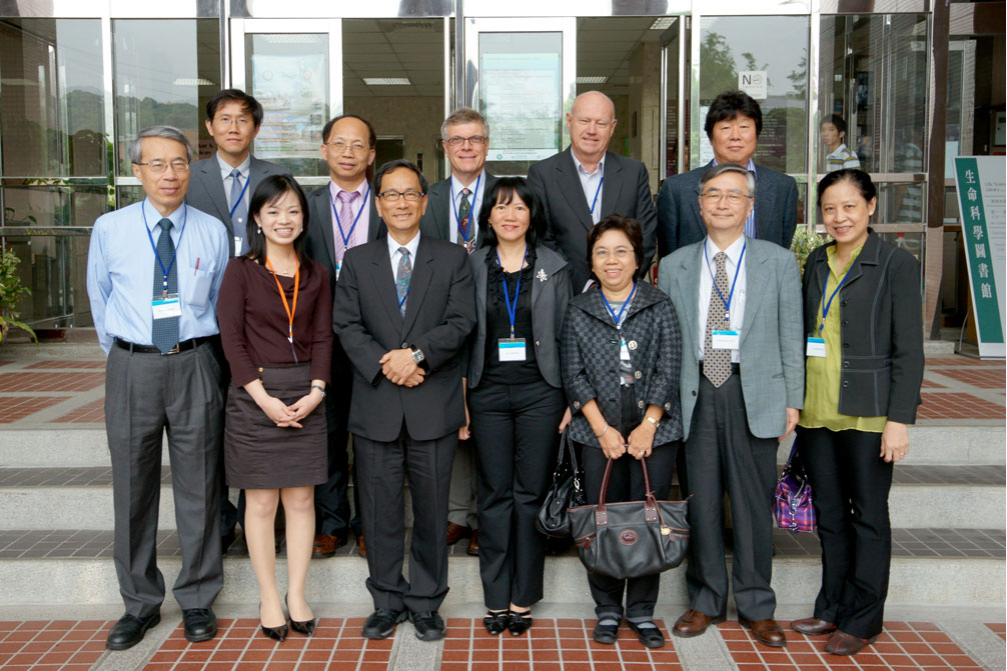
FAOBMB Executive Committee members and other colleagues pictured at the Institute for Biological Chemistry (IBC), Academia Sinica, Taipei, China, in May 2011. Back row (from left): Tzu‐Ching Meng (IBC), Yau‐Huei Wei (Taipei, China), Phillip Nagley (Australia), John de Jersey (FAOBMB Secretary General, Australia), Uhtaek Oh (FAOBMB Treasurer, Korea). Front row (from left): Ming‐Daw Tsai (IBC), Shan‐Chi Ku (IBC), Andrew H. J. Wang (FAOBMB President, IBC), Poh‐San Lai (Singapore), Piamsook Pongsawasdi (FAOBMB Fellowships Chair, Thailand), Masamitsu Futai (FAOBMB Past‐President, Japan), Siok‐Im Koh (Singapore). Image provided by P. Nagley

The planning of the scientific meetings is a collaborative venture between the EC and the Organising Committee of the event each year. This involves pre‐planning on the part of the host Constituent member with regular reporting to EC and Council of the planning for the scientific program and the budget. When the costs of air travel became less onerous around the region, especially at the start of the 2010s, EC meetings were held at which the Chairs of the Organising Committees of the next Congress of Conference would attend the EC meeting as observers, and there would often be a small scientific symposium as well. An example is the gathering at the EC meeting held in Taipei, China in May 2011 (Figure 10). The attendees from Singapore on that occasion were organisers of the 22nd FAOBMB Conference to be held in October 2011. Just 9 years later, due to the COVID‐19 pandemic, there were severe disruptions to all the face‐to‐face meetings, as discussed in a separate section below.

## 5 FELLOWSHIPS

### 5.1 FAOB(MB) travel fellowships

The commencement of FAOB scientific meetings in 1977, and their continuation on an annual basis thereafter, stimulated EC and Council in 1980 to assign a portion of their meagre funds to the provision of travel fellowships enabling young scientists from around the FAOB region to attend and participate. Preference was to be given to ‘those resident in countries where the practice of biochemistry is in the early stages of development’. In those early days the definition of ‘young’ was not specified; it was left to the applicants to self‐assign and for those participating in the selection of candidates to judge if the applicant was young enough. Under this system travel fellowships costing FAOB about $700 for each fellow were awarded. Initially there were five FAOB Travel Fellowships awarded for the 2nd FAOB Congress in Bangalore, India in 1980. The numbers per scientific meeting have remained of the order of 5–10 for most years till the present, depending on the number and quality of applicants each year.

Overall, since 1980 about 265 FAOB(MB) Travel Fellows have enjoyed to opportunity to travel to a host country for a Congress, Symposium or Conference and to participate in the scientific meeting. In the early years, applicants had to write about their research in their applications but later they needed to include the Abstract of their planned presentation at the scientific meeting. Once selected, the Travel Fellows each present their work in a poster session. In recent years, since 2013, a session has been reserved in the program at each FAOBMB scientific meeting (where there is no associated YSP event; see below) for the Travel Fellows to present a short talk on their work, in addition to their presenting a poster.

The definition of ‘young’ was finally addressed in 2003, after a set of Guidelines for FAOBMB Travel Fellows was prepared by Perumal Ramasamy (Fellowships Chair that year) and Bill Sawyer (Past‐President). These Guidelines specified an upper age limit of 40 years for eligibility for Travel Fellowships; the Guidelines became formally part of Standing Orders in 2007. This generous upper limit took into account the extended career development periods prevalent in some countries of the region, where commencement of PhD studies could be delayed, the candidates further having to spend some years in another country for their doctoral studies. These Guidelines were rewritten in 2013 to express them in the formal language appropriate to Standing Orders; a Guidelines document, as such, was retained, as it was more easily read by potential applicants. In 2021, a revision of the Standing Orders and the associated Guidelines, led by Fellowships Chair Paul Gleeson, changed the definition of eligibility to ‘registered PhD candidates and early career scientists of not more than 10 years post‐graduate experience’. This was done to normalise the eligibility for the Travel Fellowships across the FAOBMB region, although the representation of Travel Fellows from less well‐developed countries in the region had always been relatively high compared to those from well developed countries.

### 5.2 Administration and range of travel fellowships

When the Travel Fellowships were first awarded in 1980, they were administered directly by the Secretary General and Treasurer of FAOB, working within EC. In 1983, the FAOB Travel Fellowships Committee was established, as mentioned above in the Section on Governance and Administration. The full list of Chairs of the Fellowships Committee can be found on the FAOBMB website at: www.faobmb.com/about-faobmb/history-of-faobmb/. The composition of the present Fellowships Committee can be found at: www.faobmb.com/fellowships/faobmb-fellowships-committee/. The Fellowships Committee is responsible for the assessment and ranking of applications for the Travel Fellowships (and members of that Committee also participate in assessment of other FAOBMB Fellowships mentioned below).

Details of the various Fellowships currently offered by FAOBMB can be found under the Fellowships Tab of the webpage at: www.faobmb.com. In addition to the Travel Fellowships discussed above, there are several more categories. Travel YSP Fellowships are provided instead of Travel Fellowships in years when there is a YSP event associated with the annual scientific meeting of FAOBMB. These YSP events are focussed on young scientists from the FAOBMB region who assemble for a two‐day meeting (usually at a separate site from the Congress or Conference that year) involving scientific sessions, career development sessions, and activities of a social and cultural nature. The FAOBMB Travel YSP Fellowships are often supplemented by Special YSP Fellowships provided by Constituent Members, generally reserved for young scientists from that country to participate. Strict selection criteria are applied, and the many applications received for YSP Fellowships are reviewed by international selection committees that have the responsibility to maintain a high standard of participants, as well as to ensure equitable distribution of YSP Fellowships across the region. Where a YSP event in supported by IUBMB, the source of YSP participants usually covers all regions of the world, that is, from countries where there are Adhering Bodies of IUBMB (equivalent to Constituent Members in FAOBMB terms) or Associate Adhering Bodies of IUBMB.

FAOBMB Special Travel Fellowships enable young scientists from the FAOBMB region to attend Training Courses organised by IUBMB or other organisations. These are usually advertised in relation to specific events, which have included the IUBMB Advanced Summer Schools held in Delhi, India in 2014 and in Bangkok, Thailand in 2018.

FAOBMB Education Special Travel Fellowships are reserved for educationists and are discussed under the section on Education below. A further category is the FAOBMB Exchange Fellowship, introduced in 2014, which supports the travel of a young scientist to another country in the FAOBMB region to undertake training in a particular research technique or to undertake some collaborative research in that other country. They are similar to the Wood‐Whelan Research Fellowships offered by IUBMB (www.iubmb.org/activities/fellowship-programs/iubmb-wood-whelan-research-fellowships/) but the take up of Wood‐Whelan Fellowships by young scientists in the FAOBMB regions has been far greater than for the FAOBMB Exchange Fellowships.

There is a further Fellowship for more senior scientists called the FAOBMB Travel Lectureship, also introduced in 2014. This scheme enables travel for an accomplished individual from the FAOBMB region, invited by a host Constituent Member in another country, to deliver lectures and seminars in a research institution in the host country and to travel within that country to other institutions. As with the Exchange Fellowships, there has not been much take‐up of the Travel Lectureship scheme thus far.

## 6 AWARDS

FAOBMB began its program of Awards for Biochemist and Molecular Biologists of high achievement from the region in 2006, with the Young Scientist Awards. There have since been implemented another three awards, as discussed below. Details of the Awards can be found under the Awards Tab on the FAOBMB website at: www.faobmb.com together with a full list of the recipients of each Award, accessible from the dropdown menu that opens at the Award Tab.

### 6.1 Young Scientist Award

**FIGURE 11:**
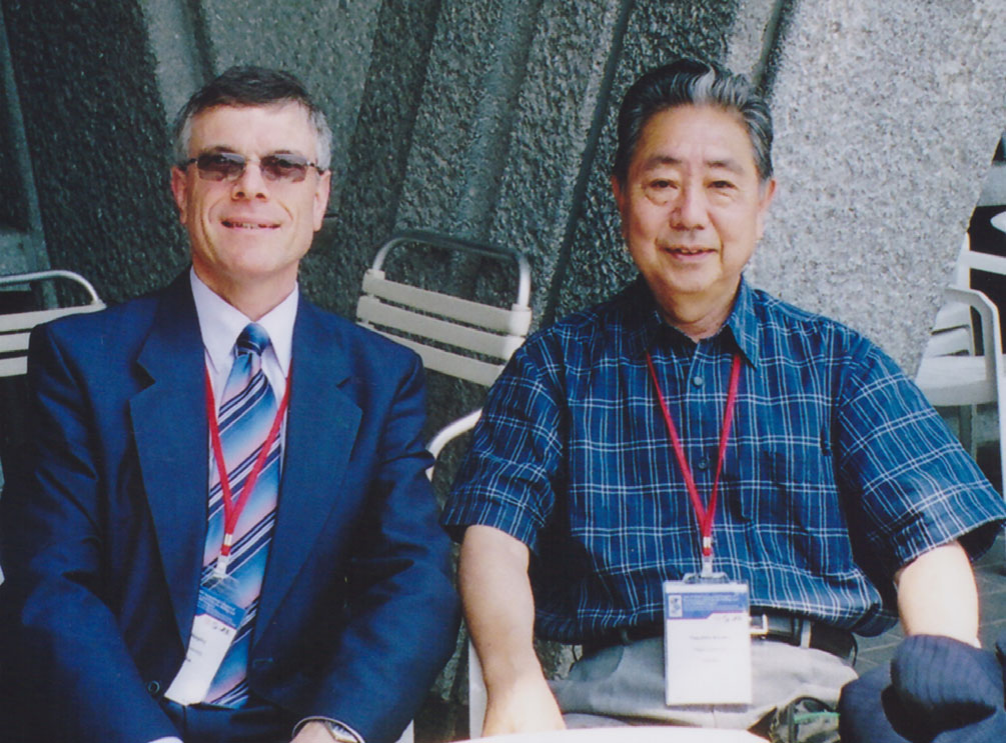
Phillip Nagley (left) and Yasuhiro Anraku (right) at the IUBMB‐FAOBMB Congress held in Kyoto, Japan, in June 2006. Image provided by P. Nagley

At its meeting on September 20, 2002 in Taipei, China, FAOBMB Council accepted a generous donation of $30,000 from Professor Yasuhiro Anraku (Figure 11), a past President and former Treasurer of FAOBMB based in Japan, as an endowment to establish the Young Scientist Awards. The terms of this award were set out that there would be two Awards every 3 years, associated with the FAOBMB Congress. One Award would be for a male scientist, the other for a female scientist, each being of <35 years of age and having membership of a Constituent Member society or group. The winners would be selected from applications judged by a newly established Awards Committee of Council, comprising specified members of EC, Committee Chairs, and the Congress Organiser for that year. The Award winners each deliver a 30‐min lecture on their research at the FAOBMB Congress. The first such pair of Young Scientist Award winners were selected to present their talks at the joint 20th IUBMB‐11th FAOBMB Congress held in Kyoto, Japan, in 2006.

The operation of the endowment is such that the principal is preserved in an FAOBMB fund and the interest pays Honoraria of $2,000 to each of the male and female Young Scientist Award winners to participate in an FAOBMB Congress (similar to the arrangements for the endowment funds for the named lectures, mentioned above in the section on Congresses). The Guidelines for the Young Scientist Award were first formulated in 2003, and appended to the Guidelines for Travel Fellowships, as first prepared that year by Ramasamy and Sawyer (see section on Fellowships, above). It was only during the extensive revisions of the Standing Orders finalised in 2014 (see section on Governance and Administration, above), that the Young Scientist Awards found their rightful place there, alongside the specifications of the other Awards, which are considered below.

### 6.2 Award for Research Excellence

During 2009, FAOBMB EC began to develop proposals for both an FAOBMB Medal and an FAOBMB Education Award, stewarded by Secretary General John de Jersey (Figure 10). These proposals were accepted in principle by Council at its meeting in Shanghai, China in August 2009, subject to appropriate financial sponsorship for these Awards being obtained. The model for these proposed Awards was based on the system utilised by ASBMB in Australia, where the detailed documentation of the regulations, and the nomination or application processes, as well as the judging, were well established (in the ASBMB case sponsorship for the Education Award came from a local Company). Following the accession to President‐Elect in 2010 of Andrew Wang (Figure 7) from Taipei, China, he was able to secure funding for these two Awards from the Yong‐Ling Foundation in his country, such that an Honorarium of $3,000 was paid to each of the Award winners, plus an additional sum of up to $2,000 for travel expenses to attend the FAOBMB Congress or Conference where the winner would present a plenary lecture. These funds from Yong‐Ling were made available from 2011 not only for these two Awards but also later for the Entrepreneurship Award introduced in 2014 (see below). In 2018, after the Yong‐Ling funding had ceased, Andrew Wang was able to secure funding from the Biochemical Technology Education Foundation, based in Taipei, China, to support these three FAOBMB Awards for the triennium beginning 2018. Such generous funding was extended once again in 2021. It remains an issue for FAOBMB to secure more permanent funding for the future for these Awards.

At its inception in 2011 at the 22nd FAOBMB Conference held in Singapore, the FAOBMB Medal was referred to as the FAOBMB Award for Research Excellence; this is the name in current usage by FAOBMB in the Standing Orders, Guidelines and other documents of the Federation. There is a process of nomination by senior colleagues of suitable scientists in the FAOBMB region (done with the knowledge of the nominee) for consideration by the Awards Committee of Council. The Nomination Form requires a description of the nominee's major scientific contributions in relation to the objectives of the Award, the significance of these contributions, an explanation as to why the nominee is especially qualified to receive the Award, and a description of the nominee's contribution where the nominee has been part of a research team or teams. The inaugural winner in 2011 was Professor Cheng‐Wen Wu of Taipei, China. The Award for Research Excellence is the most prestigious Award of the Federation and has been awarded annually since 2011 to many highly accomplished biochemists and molecular biologists within the region.

### 6.3 Education Award

Following the 12th FAOBMB Congress in Bangkok in 2012, at which the second Award for Research Excellence lecture was given, and those of the two Young Scientist awardees were delivered, a cycle of Awards was set up in which the next FAOBMB Conference included the lecture by the recipient of the Education Award, and the following Conference included that of the Entrepreneurship Award (in both such Conference years the Award for Research Excellence also took place). This cycle continues to the present.

The FAOBMB Education Award is given triennially to a biochemist or molecular biologist, in recognition of outstanding contributions to education in biochemistry or molecular biology in the FAOBMB region, with a special focus on innovation and productive outcomes in education. Candidates directly apply for this Award, rather than being nominated. Documentation of the candidate's achievements in the Application Form covers many aspects, including: teaching philosophy and methods; personal teaching performance, quality and outcomes; Biochemistry and Molecular Biology research and teaching: evidence of involvement in research (education, scientific or both); external role, peer review and influence beyond host department/institution. There are multiple domains and indicators of achievement provided in the Applicant Form under each of the aspects. The evaluation of the candidates against specific criteria is thus specific to the educational context and covers topics with much different emphasis from those set out for the Award for Research Excellence (see above). The Selection Committee for the Education Award has members with special expertise in education as well as specified members of EC and the Organiser of the Conference that year.

The FAOBMB Education Award has been presented three times, with the inaugural Award presented to Professor Jane Macaulay of Melbourne, Australia (who later went on to become IUBMB Chair of Education and Training from 2016 to 2021). All the awardees have made excellent innovative contributions to teaching and learning of Biochemistry and Molecular Biology. Their plenary lectures all have been keynote addresses for the Education Symposium at the same Conference (see section on Education, below).

### 6.4 Entrepreneurship Award

The Entrepreneurship Award commenced in 2014 and is given triennially for outstanding achievement in entrepreneurship in biochemistry or molecular biology, especially innovation and creativity in research or technology, and their translation to broader aspects, with a view to fostering leadership in this important area of the FAOBMB activities. The emphasis is, therefore, on research applied to specific outcomes, rather than the ‘pure’ research outcomes that characterise the Award for Research Excellence. That said, the recipients of the latter Award often have well developed applied aspects, especially to medical or agricultural spheres. The criteria for the Entrepreneurship Award refer to a successful scientific career, indicative of significant sustained contributions to biochemistry and molecular biology; translation of research technology to the scientific community, with demonstrations of the application to practice of innovative technologies or methods; translation to the commercial world; entrepreneurial outcomes in broader aspects of science and technology, encompassing contributions to various areas of health, agriculture or public science policy; contributions to Science and Society in broader terms, perhaps including dissemination of scientific information to the wider community, promotion of the appreciation of the relevance of science, and innovation in effective educational programs that increase the participation of school students in the study of science.

The number of applications for the Entrepreneurship Award have been much fewer than for the other Awards of FAOBMB. It is possible that the criteria are too onerous for many scientists to submit what they perceive as credible applications. Nonetheless there have been outstanding recipients of both the inaugural award in 2014, Professor Anthony Weiss from Sydney, Australia (whose achievements have been in human tropoelastin and its assembly to make three‐dimensional elastin protein biomaterials to enable human tissue repair), and the award in 2017, Professor Masatoshi Hagiwara from Kyoto, Japan (who has developed novel therapeutic methods manipulating the transcriptome with small chemicals to cure genetic diseases, with applications to cancer treatment). Unfortunately, in 2020 there were insufficient applications to make an award that year. It is to be hoped that successful scientific entrepreneurs in the FAOBMB region with the relevant achievements in biochemistry and molecular biology can be encouraged to make applications in the future.

## 7 NEWSLETTER/BULLETIN

### 7.1 FAOB News

On the foundation of FAOB, the Secretary General/Treasurer N. R. Mougdal (Figure 2) became responsible for the preparation and circulation of a newsletter. The *FAOB News* was printed in Bangalore, India twice per year and was distributed among the FAOB community through the offices of the national societies/groups. The newsletters contained information about meetings and workshops in the region (and elsewhere in the world) plus details of scientists known to be soon travelling to the region. These early issues were very important for dissemination of information concerning the nascent Federation, especially before the scientific meetings of the Federation, as such, had commenced.

**FIGURE 12:**
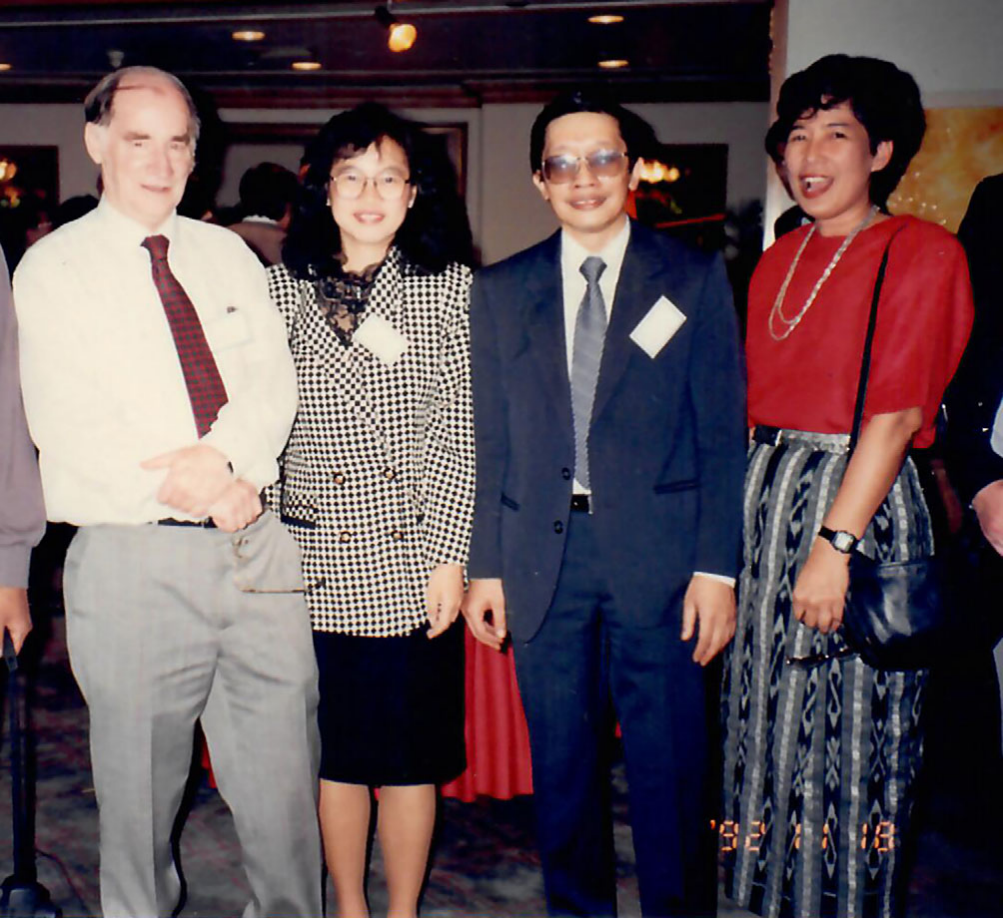
William J. O'Sullivan (Australia, Editor FAOB News), Rudee Surarit (Thailand), Jisnuson Svasti (Thailand, President), and Jariya Boonjawat (Thailand), taken at the 6th FAOB Congress, Shanghai, on 18 November 1992. Image provided by J. Svasti

**FIGURE 13:**
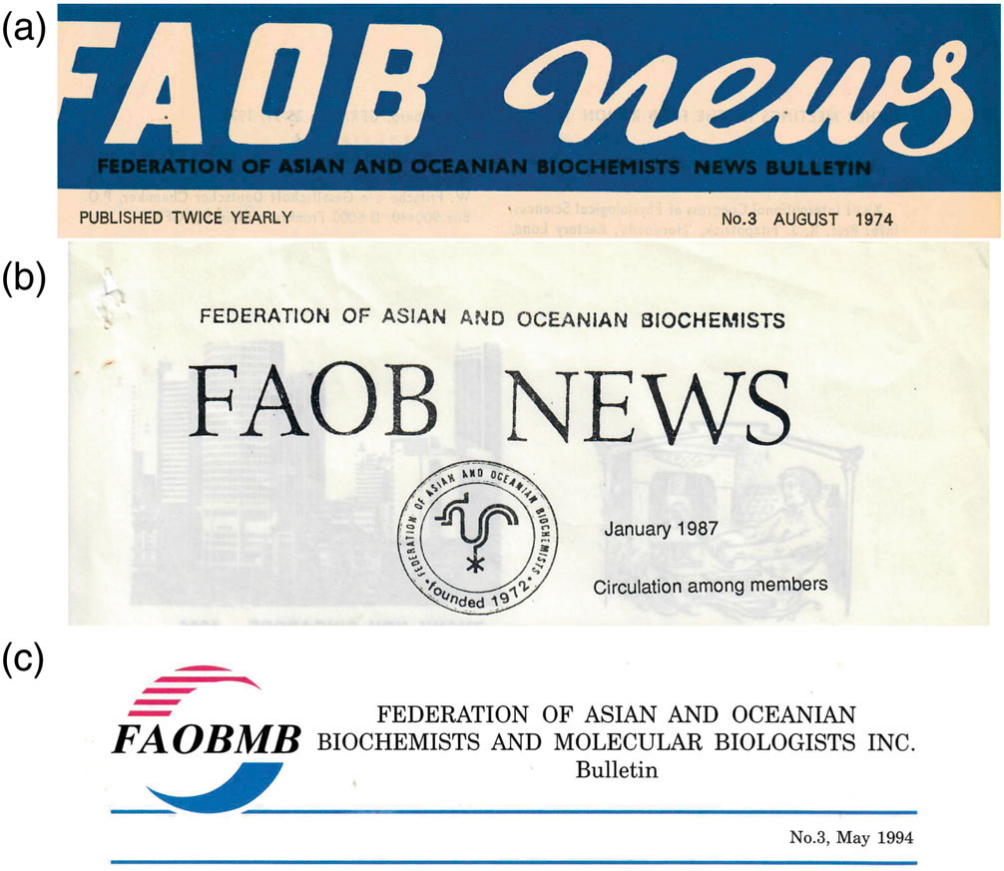
Mastheads from Newsletters and Bulletins. (a) FAOB News, August 1974. (b) FAOB News, January 1987. (c) FAOBMB Bulletin, May 1994

The production of these early FAOB Newsletters petered out after a few years and by 1983 production of the *FAOB News* had ceased. In 1983 at the 3rd FAOB Congress in Bangkok, Thailand, the President Osamu Hayaishi and Secretary General Fyfe Bygrave, together with the Fellowships Chair Takashi Murachi, approached William J. O'Sullivan from Sydney, Australia, to suggest that he act as Editor of a revived FAOB Newsletter. Bill O'Sullivan (Figure 12) agreed to take up this role, which he stayed in for the next 11 years. In contrast to early editions of the *FAOB News* prepared by Mougdal, where no logo was used in the masthead (Figure 13A) the weathercock logo appeared at the masthead of the *FAOB News* in the 1980s (Figure 13B). The news material and other information collated by O'Sullivan for the newsletter was laid out for presentation, and brilliantly illustrated, by his colleague at the University of New South Wales, Philip J. Schofield. Using his nickname Fil, Schofield included relevant witty images from a variety of classical and modern sources, including his own cartoons. The *FAOB News* of that period was printed in Sydney, and about 20 copies were sent to each delegate of Constituent Members for further copying and distribution within the various countries of the FAOB community at the time.

By 1992, O'Sullivan was finding the task of getting information from Constituent Members more than a little frustrating, exacerbated by his receiving very little feedback from colleagues in the region. As well as requesting some financial support, O'Sullivan agreed to act as Editor of the Newsletter for another 2 years until 1994, but from 1993 it was produced under the rubric of the Bulletin (see next sub‐section).

### 7.2 FAOBMB Bulletin

**FIGURE 14:**
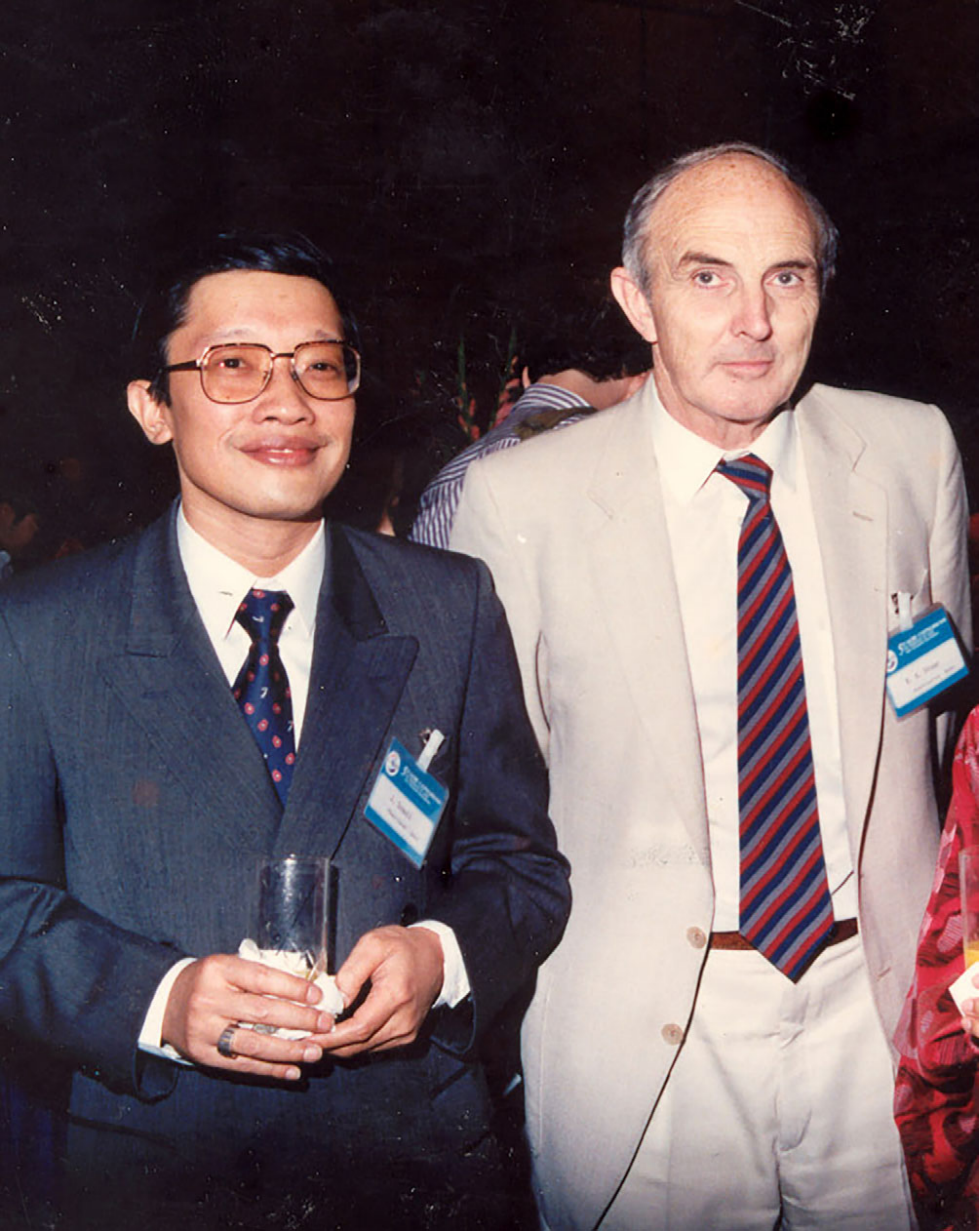
Jisnuson Svasti (left) and Bruce A. Stone (right) at the 5th FAOB Congress held in Seoul, Korea, in August 1989. Image provided by J. Svasti

In the meantime, during 1990 and 1991, Council had been debating propositions to establish an FAOB Journal (see section on Journal, below). Part of those discussions centred on whether a purely research journal was envisaged or a publication containing both research articles as well as news and other information about FAOB. Council leaned towards the notion of a purely research journal, and decided that a separate Bulletin could be established, eventually to replace the *FAOB News* but with more substantial content. Thus, Council determined in 1991 that a committee would be set up, with Bruce Stone from Australia as Chair, to consider the publication of an FAOB Regional Bulletin. In 1992, Council decided to proceed with such a publication, with some initial funds provided by ASBMB. The Bulletin was to include advertisements from the Special Members and others, which would contribute to the costs of publishing and distributing this publication. Bill O'Sullivan became Editor of the *FAOB Bulletin* in 1992; for this publication Bruce Stone (Figure 14) compiled the articles and prepared a digital version, which was then saved to a Compact Disc format. The disc for each issue was sent to the Production Editor Wing‐Ping Fong in Hong Kong, where it was printed commercially. The masthead of the *FAOBMB Bulletin* (Figure 13C) contained the new FAOBMB logo when published in 1994.

When Stone became Editor of the *FAOBMB Bulletin* after 1994, the number of copies became very large, as Council had resolved that every individual member of the Constituent Member national societies/groups should receive one copy, supported by FAOBMB. This amounted to print runs in Hong Kong of over 20,000! To try and save on postage to individual members within Japan, JBS agreed to bind the FAOBMB Bulletin inside its *Journal of Biochemistry* (*Seikagaku*), which was mailed out individually to 14,000 recipients in Japan. This plan eventually faced technical and other organisational difficulties. The distribution costs of the remaining 6500 copies to the 17 other countries in the FAOBMB community were very high.

In 1995, Bruce Stone advocated that it would be most efficient to place the electronic version of the Bulletin on an FAOBMB webpage (he recommended that hosted by his own institution, La Trobe University, in Melbourne; see section on Webpage, below). Recognising that these technical developments were not yet amenable to many individual scientists in the FAOBMB region, Council insisted on the production of printed hard copies for all. But budgetary problems beset the *FAOBMB Bulletin* when the revenue stream from advertising began to dry up and the costs of production and postage increased. The Bulletin was eventually reduced in size from 8 to 4 pages to save such costs, but it was still an uphill battle for Stone both administratively and financially. In 1998, Council finally agreed that the Bulletin should be placed on the FAOBMB webpage, that by now having been set up in Hawaii by Richard Guillory (see section on Webpage, below). But in 1999 Stone went on Sabbatical leave in Denmark and no Bulletin was prepared that year.

With the impending move of the webpage to Singapore, following the election of Hoon‐Eng Khoo to the Secretary General position, Council decided in 1999 that the contents of the Bulletin be now incorporated into homepage of the Federation. It was thus the development of global internet technology (the World Wide Web), and its almost universal accessibility by the beginning of the new Millennium, that led to the demise of the Bulletin. Information such as notices of meetings, fellowships, and other news could now be readily disseminated from the FAOBMB webpage to the whole FAOBMB community very efficiently and much more cheaply than before.

## 8 JOURNAL

### 8.1 The dream of the Federation from its inception

During meetings in 1971 and 1972, before the foundation of FAOB, many of those involved were interested in the possibility of publishing a Journal of the Federation in Japan, in place of or together with the flourishing *Journal of Biochemistry* (*Seikagaku*). They considered that this would help promote biochemistry in the region under a regional ‘flagship’ publication and possibly bring income to the Federation (as achieved with FEBS). A potential title, *FAOB Letters* was discussed, but these ideas were put ‘on hold’ after a meeting in July 1972, when a review of the costs by Takashi Murachi revealed that an investment of $20,000 would be required, which was well beyond the resources of the nascent Federation.

The lack of funds in the Federation to make a capital investment in its own research journal was a major barrier in development for 25 years. Even when the FAOBMB Journal was established in 1997 (see next sub‐section below) as the *Journal of Biochemistry, Molecular Biology and Biophysics* (JBMBB), FAOBMB had no financial equity in the Journal. This led to significant problems later when the publisher, who owned all aspects of the Journal, decided to cease publication at the end of 2002, because the subscription take‐up was so poor that production of JBMBB was causing too great a loss for the company to bear. These aspects are considered in the relevant sub‐section below.

For the next few years until 1988, discussion took place at several meetings of FAOB Council, but apart from proposals to have news about FAOB included in IUB journals such as *Trends in Biochemical Sciences*, circulated worldwide, there was no progress in establishing an FAOB research journal. Interestingly, an international journal publishing short research reports, *Biochemistry International*, was established by IUB, based in Australia within the FAOB region, and published by the Sydney Office of Academic Press, with Tony Linnane as Chief Editor. By 1988, this new rapid‐publication journal (modelled on *Biochemical and Biophysical Research Communications*) had already become a vehicle for publication of short research articles from the FAOB region and other regions of the world. However, neither *Biochemistry International* nor its successor *Biochemistry and Molecular Biology International* (renamed in 1993) ever reached the desired high ranking. But in 1999 this rapid‐publication journal eventually morphed into a new more sophisticated format as *IUBMB Life* that after another decade, greatly improved its ranking as judged by Impact Factor.^9^ The modestly successful operations of *Biochemistry International* by 1990 did not diminish expectations that FAOB could successfully establish its own journal.

### 8.2 Establishment of JBMBB

In 1989, at its meeting in Seoul, Council voted narrowly (7 vote to 6) to launch a research journal with research papers and scientific reviews. A Working Party was formed and in 1990 recommended to Council that an FAOB Publication Committee should be established, which would report back to Council in 1991. After considering wide ranging opinions of Constituent Members, from a survey carried out by Secretary General Kay Hoon Lee (also a member of the Publications Committee), recommendations were made both to establish a Journal and to improve the Newsletter or Bulletin to include more scientific and technical content. A new Working Party was set up to ‘flesh out’ these recommendations. Thus in 1992, at the Council meeting in Shanghai, changes were made to the *FAOB News* (as discussed in the section on Newsletter/Bulletin, above). Significantly, this meeting also authorised the FAOB Journal Committee to formulate a business plan for publishing a ‘quality journal’ and, after consulting publishing houses, to report back to Council. Richard Guillory (Figure 5), the delegate to Council from Hawaii, emerged as the Chair of that Committee and became a strong advocate for the new Journal.

In his report for Council in 1993, Guillory referred to himself as the Managing Editor Designate and recommended that a prominent Japanese scientist, Ikuo Yamashina, should become the Editor‐in‐Chief. Some discussions had commenced with various publishing houses for the proposed Journal, which did not yet have a finalised title. Other options for publishing were considered, such as ‘piggy‐backing’ onto existing journals like *Seikagaku* of JBS or considering an IUB journal that might be conceded to FAOBMB, but no workable solution was found. At that 1993 Council meeting, Guillory brought a draft Contractual Agreement with Harwood Academic Publishers (a subsidiary of Gordon & Breach). Council was not satisfied with the proposed contract but wanted the preparations for a Journal to continue. Guillory was requested to obtain further information and to ask Harwood to adjust some terms of the proposed Contract; he was asked for more specific plans before Council could decide on the matter.

Guillory reported back to Council in 1994, on discussions with more publishers, yet continuing to hope that the Harwood contract could be modified to become acceptable. At this meeting, Council expressed the view that the Federation would like to have ownership of the Journal and copyright matters (and recorded its willingness to spend money to initiate the Journal—but FAOBMB financial support seemed to be extended only to support Guillory in his negotiations, rather than investing in the publication). Council authorised EC, the Editor‐in‐Chief and the Managing Editor to negotiate these matters with publishers and to make a final decision on which one to proceed. Council also asked Yamashina and Guillory for specific ideas for the Title of the Journal, which was suggested to be along the lines of ‘Journal of Biochemistry and Molecular Biology’.

In 1995, Yamashina reported to the Council meeting in Sydney, that after much discussion, a contract (Publishing Agreement) with Harwood Academic Publishers had been signed by the President Ram Bhagavan (on behalf of FAOBMB), Yamashina as Editor‐in‐Chief and Richard Guillory as Managing Editor. Significantly, the publisher owned the Title that was stated in the Publishing Agreement to be the *Journal of Biochemistry, Molecular Biology and Biophysics*. Since both Bhagavan and Guillory were based in Honolulu at the same institution, discussions and negotiations would have been facilitated.

By 1996, planning for the Journal was well underway and a mock‐up of the front cover of JBMBB was presented to the Council meeting, which bore the statement ‘*The Official Journal of the Federation of Asian and Oceanian Biochemists and Molecular Biologists (FAOBMB)*’. This must have settled the concerns of some of the EC and delegates, expressed in previous meetings of Council, whether FAOBMB would be acknowledged as having a sponsorship arrangement for the Journal. Nevertheless, the publisher held the financial control of the Journal. At the meeting of Council in Manila in 1997, it was proposed by the President Yasuhiro Anraku that the roles of the Editor‐in‐Chief and the Managing Editor should be included in the Standing Orders, but it was not until the Council meeting in Singapore in 2001 that the relevant clauses were included.

The initial plan was for the journal to publish relatively short research reports (termed Short Communications) and occasional mini‐Reviews written at the invitation of the Chief Editor. An Editorial Board was set up, comprising of well recognised scientists from both the FAOBMB region and the rest of the world (notably the United States of America and the United Kingdom). There was an Advisory Board consisting of the 19 delegates to Council in 1996, to indicate the ‘attachment’ of FAOBMB Council to the Journal.

**FIGURE 15:**
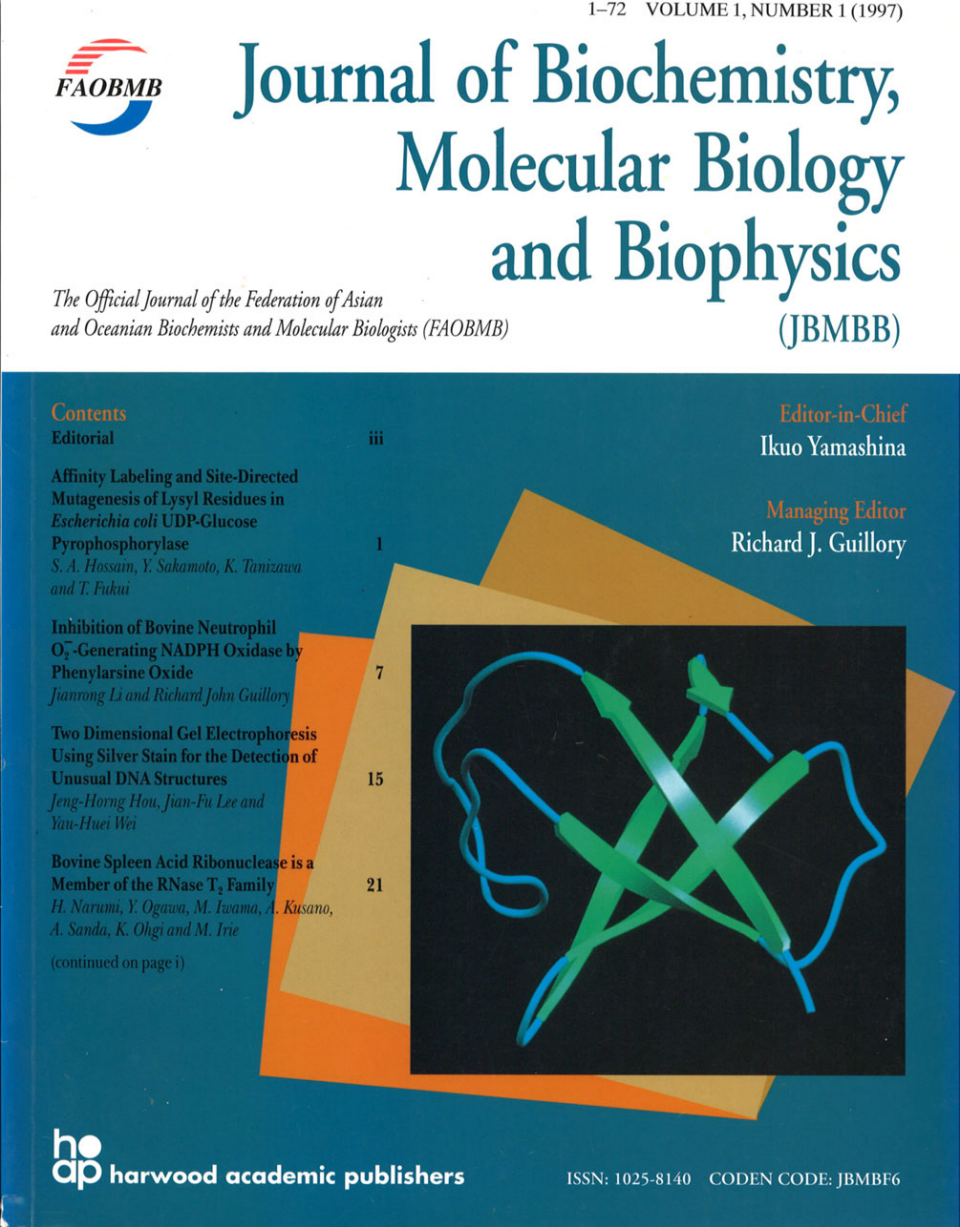
Front cover of the first issue of the FAOBMB Journal (JBMBB) published in August 1997

Publication of the first issue took place in August 1997, the front cover of which is shown in Figure 15. This issue contained, in addition to 10 research papers, an Editorial from FAOBMB President Anraku, and short Introductions from the Editor‐in‐Chief Yamashina and Managing Editor Guillory. Publication continued steadily with four issues each in Volumes 1 (1997–1998) and 2 (1998–1999), but only 3 issues in Volume 3. As the number of papers submitted increased, JBMBB was published bimonthly from 2000 to 2002. About 75% of the published Short Communications came from the FAOBMB region. In addition, invited reviews were published from time to time. Guillory envisaged increasing both the number and proportion of submitted research articles from the rest of the world, and moving to monthly publication after 2001, but his plans were thwarted by the very poor uptake of subscriptions.

As early as 1998, the dearth of subscriptions was brought to the attention of Council by Guillory and despite many efforts to improve subscriptions and to improve the visibility of the Journal (for example by increasing and widening the Editorial Board), the financial position of the journal in the hands of Harwood Academic Publishers deteriorated. It was the takeover of Gordon & Breach (who owned Harwood) by another publisher Taylor & Francis in February 2001 that sealed the fate of JBMBB. At the Council meeting in Singapore in November 2001, Guillory reported that the new publisher had recognised from their financial audit that the situation of JBMBB was not at all healthy, there being only 13 academic and 30 editorial subscriptions. Guillory warned that curtailment of the Journal would occur unless the financial situation was turned around. But this subscription issue was not something that Council could remedy quickly.

In an email to the Managing Editor Guillory and Editor‐in‐Chief Yamashina (copied to the FAOBMB President Bill Sawyer) dated 26 June 2002, the Editorial Director for Science Journals at Taylor & Francis, Richard Steele, wrote that JBMBB would cease publication at the end of 2002. The reasons for the termination of publication of JBMBB were stated in the email to be that JBMBB had failed to ‘establish a regular publication cycle’ and had failed to achieve ‘an appropriate level of subscriptions’. The email notified the parties that they would be released from all responsibilities under the Publishing Agreement with Gordon & Breach/Harwood Academic Publishers. This was an essentially commercial decision to terminate publication and this position was maintained by Steele even after communications from both Guillory and Sawyer. One concession made by Steele to Sawyer in October 2002 was that the rights to the Title of the journal would be passed to FAOBMB, after signature of transfer documents, which took place on 1 January 2003.

### 8.3 Efforts to resuscitate JBMBB

The loss of JBMBB was a bitter blow to many associated with the Federation who had dreamed of a Journal as an indispensable component of a thriving regional organisation. Others took a more sanguine view, recognising the enormity of the task required to sustain an FAOBMB Journal of very high quality, in the face of fiercely competitive publishing. It was further recognised by many in FAOBMB that the Federation did not have a financial base to invest directly to establish and maintain its own Journal, so FAOBMB only had an advisory role in JBMBB, even though it was labelled as the ‘Official Journal of FAOBMB’.

In the ensuing years, some attempts were made to resuscitate an FAOBMB Journal. Council supported initiatives to contact potentially interested publishers and empowered EC to pursue such discussions with reputable publishers. As late as 2009 some possible leads were being explored, but nothing came of them. Thus, the Minutes of the Council meeting held in 2010 in Melbourne reported that EC no longer wished to pursue the matter of an FAOBMB Journal. EC reported that they ‘felt that only a high quality journal would be appropriate and that FAOBMB had insufficient capital and personnel resources available to achieve a high‐ranked journal at this time’.

## 9 WEBPAGE

### 9.1 Early versions of the webpage

The first attempts to set up a webpage for the Federation arose in 1995, when Bruce Stone as editor of the FAOB Bulletin realised that it would be much more economical for distribution of the Bulletin if it were presented in electronic form on a suitable webpage. Accordingly, he arranged for such a website to be hosted at La Trobe University, in Melbourne, where he was based. This website did host the Bulletin for another 4 years, but for most of that period hard copies were still distributed across the region, because access to the internet was not well provided in many countries of the FAOBMB region and many individual scientists and students did not have devices able to access for on‐line reading, or to download files and print the Bulletin for themselves.

In parallel, Richard Guillory in Hawaii, who was the designate editor of the FAOBMB Journal (see section on the Journal, above) set up in the mid‐1990s a webpage hosted by an internet provider that he had access to in his region, Lava.Net. The URL he established was originally in his own name, but later became designated as an FAOBMB webpage. This was intended to house more general information about FAOBMB. Jisnuson Svasti in 1997 provided Guillory with the information that was to be placed on the webpage, which covered foundation, membership, administration, scientific meetings, fellowships, newsletter, journal, together with historical lists of Constituent Members, EC members, and Congresses and Symposia.

In 2000, with the election of Hoon‐Eng Khoo (Figure 6) as Secretary General, the responsibility for the webpage was taken up by her and the FAOBMB website was then hosted at the National University of Singapore, where she was based. At the Council meeting in November 2001, the Rules of FAOBMB were amended to add a new line to the statement of duties of the Secretary General, namely: ‘be responsible for the maintenance of the World Wide Web site of the Federation and for keeping it up to date’. This new sub‐clause did not preclude the Secretary General from assigning others to carry out the technical work of loading the relevant material onto the FAOBMB webpage and ensuring its proper presentation and accessibility to users anywhere in the world. The Secretary General was, however, required to take responsibility for the content of the webpage and ensuring it was updated appropriately from time to time. Thus, when John de Jersey (Figure 10) was elected Secretary General to take up office in 2006, the FAOBMB webpage had to find a new home. Working in Brisbane, Australia, de Jersey initially arranged with ASBMB that an FAOBMB webpage could be added to the already well‐developed webpage of that Society. The webmaster of ASBMB at the time, Tristan Wallis, was also based in Brisbane so that the technical aspects of loading content for FAOBMB were readily achieved. But the content of the website did not evolve much past what it had earlier been, while previously hosted in Hawaii and in Singapore.

### 9.2 Modernisation and expansion of the webpage

By today's standards, all these earlier webpages of the Federation were not well developed and by 2010 were not serving the purposes of the Federation to an optimal extent. Thus, when Andrew Wang joined FAOBMB EC as President‐Elect in 2010 he undertook to have the website transferred to Taipei, China, where he was based (Figure 10). The modernised FAOBMB website was hosted by Academia Sinica, where it was set up in the extended multi‐page structure that can still be found in the present website at www.faobmb.com; however, the URL of the first dedicated FAOBMB website was www.faobmb.net (which is no longer relevant). The key features of the Taipei‐hosted website were Tabs across the top of the webpages that listed topics such as About, Awards, Fellowships, Contacts. These Tabs contained dropdown menus enabling access to subsidiary pages. There were news items and upcoming events highlighted on the homepage; such details were found in the form of Posts on the website containing the relevant information. Technical staff at Academia Sinica carried out the loading of material and maintenance of the site.

Liaison by the Secretary General John de Jersey until 2011, and later by his successor Phillip Nagley from 2012, took place via Dr Shan‐Chi Ku, personal assistant to Andrew Wang (Figure 10). The processes of communication and uploading new material were occasionally delayed and subject to errors beyond the control of the Secretary General. Nonetheless, the overall outcome was well worthwhile and a credit to Andrew Wang and his team, in that the Federation now had a dynamic and visually pleasing webpage that was regularly updated with information encompassing activities of the Federation. By this time, at the end of the first decade of the 21st Century, scientists in the FAOBMB region and beyond all had access to devices, either desktop or hand‐held, which could efficiently get information from the webpage. Thus, by 2011, the announcement of events, awards, fellowships and so on, which required applications to be submitted by specified deadlines were first circulated by email from the Secretary General to the EC and Delegates who would then circulate the email to all members of the national Societies or Groups that constituted FAOBMB. Links to the relevant section of the FAOBMB webpage enabled access to detailed information about scientific meetings, together with Guidelines and Application Forms for Awards and Fellowships.

In 2015, under the stewardship of Secretary General Phillip Nagley, the FAOBMB webpage was transferred to Melbourne, Australia, where it was set up by the abCreative company (which continues to provide technical support). The FAOBMB webpage was reconfigured to go onto the WordPress platform, which made it readily editable by the Secretary General or others who are not specifically technically trained in writing script for webpages. At this transition point, further Tabs were added including those for Education and the Equality Policy of the Federation; Events were also included in the relevant WordPress module enabling their listing on the webpage separate from other news items. For technical reasons it was not possible to continue with the previous URL of www.faobmb.net so the new FAOBMB webpage is at www.faobmb.com (some unknown person had already registered the URL www.faobmb.org for themselves!). It can be noted here that the Minutes of the Council meeting in Dunedin, New Zealand in late 1999 record the prophetic remark of the then Secretary General: ‘Qi‐Shui Lin suggested that the homepage address of the Federation should be www.faobmb.com’. This came to pass 16 years later when the new webpage was launched in early 2016!

## 10 EDUCATION

When FAOB was founded in 1972, Education in Biochemistry was not included in the stated primary purposes of the Federation (as mentioned above in the section on Governance and Administration; see sub‐section on Purposes of the Federation). Thus, for the first two decades of the Federation, sessions on education were rarely incorporated into scientific meetings of FAOB, with two notable exceptions. In 1983, the 3rd FAOB Congress in Bangkok, Thailand included a symposium on Biochemical Education, in which two of the leaders of education in the IUB community were speakers., These were Ed Wood (United Kingdom) and Frank Vella (Canada), together with other international and local educationists in Biochemistry. This set the stage for future generous support by IUB(MB) for educational activities in the FAOB(MB) region. The 6th FAOB Congress held in Shanghai China in 1992, likewise included a symposium on Biochemical Education. That said, from its inception FAOB had been keen to sponsor various research and technical training activities in the region, as opposed to dealing with undergraduate education.

Around 1997 (the exact date is uncertain to the authors) FAOB set up an Educational Committee as a sub‐Committee of Council, the inaugural Chair being Ram Bhagavan, who had just been Past President. This led to an increased ‘presence’ of educational events such as workshops or symposia on education in biochemistry and molecular biology associated with scientific meetings of the Federation. Thus, there was a Satellite Meeting on Biochemical Education at the 13th FAOBMB Symposium held in Manila, Philippines in 1997, the program for which included two IUBMB leaders Bill Whelan (President, from United States) and Leopoldo de Meis (Education Chair, from Brazil). A Workshop on Biochemical Education took place at the 8th FAOBMB Congress in Kuala Lumpur, Malaysia in 1998. Two Educational Symposia supported by IUBMB took place at the 14th FAOBMB Symposium held in Dunedin, New Zealand in 1999. The Education Symposium at the 15th FAOBMB Symposium in Beijing, China in 2000 focussed on graduate education and training at the PhD level, also supported by IUBMB with a keynote presentation by Ed Wood.

Thereafter, education events of one sort or another regularly have regularly taken place at scientific meetings of the Federation over the past 20 years, almost without exception. These symposia/workshops (and other Education Conference events; see below) have been organised or encouraged by a succession of enthusiastic Chairs of the FAOBMB Education Committee (as it became known in 2004), whose names are shown in Table 3 and listed chronologically on the FAOBMB website at: www.faobmb.com/about-faobmb/history-of-faobmb/.

The importance of these educational events, be they Satellite Meetings, Symposia or Workshops, to local communities of Biochemists and Molecular Biologists within the FAOBMB region cannot be over‐estimated. Indeed, even after two of the scientific meetings of FAOBMB were cancelled, the Education Symposia funded by IUBMB went ahead at a deferred date. That of the cancelled 23rd FAOBMB Conference that was to be held in Dhaka, Bangladesh in December 2013 was eventually held in June 2016, although none of the international speakers at the originally planned event could attend (the speakers in 2016 in Dhaka were mostly expatriate Bangladeshis). Again, after the 28th FAOBMB Conference scheduled in Colombo, Sri Lanka had been cancelled in 2020 due to the COVID‐19 epidemic, a very successful deferred but virtual Education Symposium was held in July 2021. A wide array of international and local presenters took part, and there were well‐organised breakout on‐line discussion groups. The total number of participants was estimated at 500 (from the number of logged‐in devices), many more than would normally attend an in‐person FAOBMB educational event.

FAOBMB has also been involved in the sponsorship of more specialised training courses or ‘stand‐alone’ educational events. After several years of planning for a Training Course in Bioinformatics, led by Tin Wee Tan from Singapore, for which various locations in the FAOBMB region were considered, this course was eventually held in November 2005 at Lahore, Pakistan, just before the 18th FAOBMB Symposium held in that city. The course included direct video links to Singapore where lectures were delivered remotely to the participants in the Training Course. There was another Workshop, on problem‐based learning, organised by Education Chair Hoon Eng Khoo, and an Education Symposium on the same topic, both of which were part of the FAOBMB scientific meeting in Lahore.

A further Bioinformatics Workshop was held in Hanoi, Vietnam in August 2007, also led by Tan. The participants in Hanoi were able to remotely watch the proceedings of the International Conference on Bioinformatics held in Hong Kong during the days following the Workshop. That same year in June, the Education Symposium at the 19th FAOBMB Symposium in Seoul, Korea had focussed on Biotechnology Education.

Vietnam became particularly active in such educational events, with a second Workshop on Bioinformatics and Computational Biology held in 2008, plus another workshop on more general aspects of Biochemical Education that same year at which several educationists from Australia participated. A further workshop on general Biochemical Education was held in Hanoi in 2011, with two more educationists from Australia. The delegate to FAOBMB Council from Vietnam, Professor Pham Thi Tran Chau was central to the organisation of these events.

At a broader level, following the first IUBMB Education Conference held in Rehovot, Israel in 2017, at which there were several participants from the FAOBMB region, the second such IUBMB Education Conference was organised by FAOBMB Education Chair Gracia F.B. Yu in Manila, Philippines in November 2019. Reports on the above (and other) Education Events, including those supported by IUBMB, can found on the FAOBMB webpage at: www.faobmb.com/education-and-training/iubmb-educational-activities/.

In 2017, FAOBMB increased the range of its Travel Fellowships with the introduction of the FAOBMB Education Special Travel Fellowships to enable educationists from the FAOBMB region to travel to international meetings or training courses. The first such Education Special Travel Fellowship was awarded for travel to Israel for the First IUBMB Education Conference in 2017. A further four were awarded for the 16th FAOBMB Congress held as a virtual meeting based in Christchurch, New Zealand in 2021 (see section on the Impact of COVID‐19, below). The criteria for the Education Special Travel Fellowships are broader than for regular Travel Fellowships and allow for minimal qualification at the Master of Science level plus relevant experience (rather than PhD, as such), as it is recognised that some very effective educationists may not have PhD qualifications. Aside from the regular Travel Fellowships, the other Fellowships of FAOBMB, namely, Exchange Fellowships and Travel Lectureships, have provision for them to be awarded to educationists as well as research scientists.

## 11 SPECIAL PROJECTS OF FAOB(MB)

Over the years, despite its relatively limited funding base, the Federation has extended support to needy Constituent Members, either to enhance development of countries where there are clear shortfalls in resources for biochemistry and molecular biology or in response to specific situations. A few examples are provided here.

FAOB considered in its early days that provision of textbooks in biochemistry was an issue for many of the poorer countries in the region. Efforts were made to collect recent editions of such textbooks and steps taken to distribute them to particular countries in need. When the Education Committee was established in the late 1990s, a revitalised project was initiated to distribute textbooks. Other efforts were made to respond to requests for disused equipment from individual Constituent members, but these were hampered at many stages by logistical matters of acquisition, transportation, and governmental regulations.

Even when there were weather‐induced disasters, such as the cyclone that devastated Myanmar in May 2008 it was not at all easy for FAOBMB to respond to the plea from the delegate Professor Dr Myo Win for support to restore the equipment, reagents, books, and teaching aids that were lost in the cyclone. Eventually, an arrangement was made for some equipment, especially computers, to be sent to Myanmar from regular commercial suppliers with the invoices to be sent to FAOBMB for payment.

A further specific development project to which FAOBMB contributed during 2012–2014, was the establishment of a laboratory for teaching biochemistry and molecular biology at the recently founded Asian University for Women in Chittagong, Bangladesh (at which a previous Secretary General of FAOBMB, Hoon‐Eng Khoo, had become a senior academic). The funds were transferred via the Asian University for Women Support Foundation in the United States of America and were provided only on strict auditing of expenditure on equipment and supplies specific to biochemistry and molecular biology education.

## 12 IMPACT OF COVID‐19

COVID‐19 has influenced our lives, including education and research, throughout the world since January 2020. Our activities have been restricted for more than 2 years, both by governmental rules and regulations and by our appropriately cautious behaviour, to prevent the spread of Coronavirus. FAOBMB members have tried to adapt to this ‘new normal’ and we continue to undertake our various activities in the biochemistry and molecular biology field as best we can.

Although we cannot predict the situation in the next few months, as it is changing in many different countries with the appearance of new variants of COVID‐19, the capacities and adjustments necessary to end the pandemic can and should lay the foundations for a future in which the world is prepared to prevent, detect, and respond to pandemic threats. During the COVID‐19 pandemic, academic communities have experienced many changes. Research laboratories have not always been able to open, leading to abrupt changes in hands‐on research and training. Ironically, this has led in many cases to pieces of good research work being written up for publication during closures and lockdowns, which might otherwise have been delayed in the face of continued experimentation! Colleges and Universities have been likewise subjected to limited interactions for face‐to‐face learning, and many classes have been taught remotely. This has led to a huge surge in on‐line education, much generated under stress with short timelines, placing heavy workloads on faculty as well as students. Much of the on‐line teaching and learning has remained after the closures and lockdowns were released in the past months, and many creative new ways of delivery of courses on‐line have been introduced.

**FIGURE 16:**
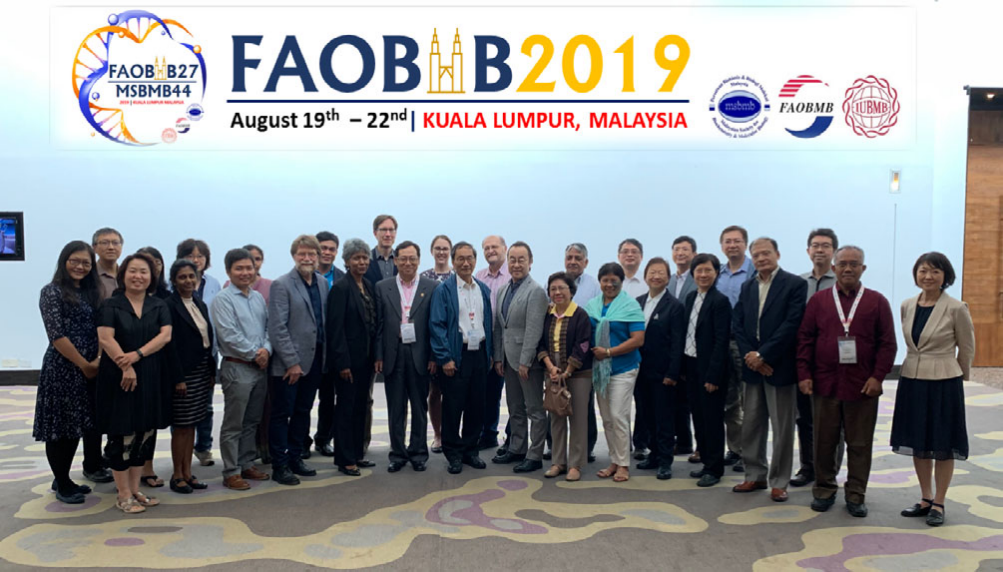
FAOBMB Executive Committee, Delegates to FAOBMB Council, and Observers at the Council meeting held in Kuala Lumpur, Malaysia, on 18 August 2019. From left: Yang‐Mooi Lim (Malaysia), Anthony Ho Siong Hock (Observer from Conference Organising Committee, Malaysia), Hyunsook Lee (Korea), Kyu Kyu Maung (Myanmar—*partially obscured*), Sharmila Jayaseena (Sri Lanka), Shannon Au (Observer as Treasurer‐Elect, Hong Kong—*partially obscured*), Le Tien Dung (Vietnam), Lohini Athithan (Observer, Sri Lanka—*partially obscured*), Paul Gleeson (Fellowships Committee Chair, Australia), Francisco Heralde III (Philippines), Sheila Nathan (Secretary General, Malaysia), Wayne Patrick (New Zealand), Zengyi Chang (FAOBMB President, China), Monica Gerth (Observer, New Zealand), Andrew H.J. Wang (Honorary Member of FAOBMB as Past‐President, and IUBMB President), Terrence Piva (Australia), Akira Kikuchi (President‐Elect, Japan), Piamsook Pongsawasdi (Treasurer, Thailand), M. Waheed Akhtar (Pakistan), Gracia F.B. Yu (Education Committee Chair, Philippines), Dong‐Yan Jin (Hong Kong), Mary Waye (Observer, Hong Kong), Fang‐Jen Lee (Taipei, China), Tuangporn Suthiphongchai (Thailand), Nei‐Li Chan (Observer, Taipei, China), Tilak R. Shrestha (Nepal), Wen‐Shan Yew (Singapore), I Made Artika (Indonesia), Mayumi Nakanishi‐Matsui (Japan). Image provided by Anthony Ho

The major ‘core business’ activities of FAOBMB, namely, face‐to‐face scientific meetings, were abruptly halted in early 2020. COVID‐19 caused disruption to the planned schedules of the Conferences and Congresses of the Federation, continuing until at least late 2022. As indicated above in the section on Congresses, Symposia and Conferences, these scientific meetings aim to promote the sharing of insightful experiences among participants and increase the global visibility of FAOBMB in the biochemical and molecular biological research landscape. The last such scientific meeting of the Federation before COVID‐19 arrived globally was the 27th FAOBMB Conference held in Kuala Lumpur, Malaysia, from 19 to 22 August 2019, which included a YSP and a Career Development Forum. The general theme of the Conference was ‘Biomolecules: Networks and Systems’ with a major symposium stream devoted to ‘Mosquito‐borne illness’. The meeting, which received IUBMB support (see section on section on Congresses, Symposia and Conferences, above), attracted over 350 delegates from 36 countries, and assembled an excellent program, including 10 Plenary and Award lectures, 18 current sessions, and 102 posters. In addition to science and international communication, participants really enjoyed culture, food, and atmosphere in Malaysia (Figure 16). All this epitomised the full flavour, so to speak, of past and current FAOBMB scientific meetings.

However, in the following year it was most unfortunate that the 28th FAOBMB Conference, scheduled to take place in Colombo, Sri Lanka in June 2020, had to be cancelled due to the COVID‐19 pandemic and the consequent travel restrictions imposed by many countries. As mentioned in the section on Education above, an on‐line IUBMB‐funded Education Symposium was held in July 2021.

In 2021, although it had been hoped that the COVID‐19 pandemic would have subsided well before the 16th FAOBMB Congress was to be held in Christchurch, New Zealand, from 22 to 25 November, travel restrictions to New Zealand came into force in mid‐year 2021 and severe restrictions on gatherings in New Zealand itself were introduced. The heroic efforts of the Conference Organiser, Wayne Patrick, together with his committee, and staff from the contracted company (Composition Limited in Christchurch), enabled the first fully on‐line Congress of FAOBMB to take place with spectacular success. Regardless of all the changes brought by COVID‐19, the vision, policy, and aim of that FAOBMB Congress remained unchanged with the scientific program including 15 plenary lectures, 50 parallel sessions, and over 300 electronically presented posters. The local organisers did a great job to organise the virtual congress, including pre‐recorded talks, virtual posters, and on‐demand delivery of contents. This new style of scientific meeting succeeded beyond the expectations of most participants in communicating science and engaging with other scientists. The tripartite Education Symposium/Workshop was especially topical as it had presentations on educational on‐line delivery and sessions on virtual reality for presenting and examining protein structures, including live demonstrations to the on‐line audience.

Concerning the 29th FAOBMB Conference, scheduled to be held in October 2022 in Shenzhen, China, this was planned for much of 2022 as a hybrid meeting, with local Chinese participants in‐person and international participants on‐line. However, late changes in regulations locally concerning COVID‐19 have caused the meeting to be adjusted to an online‐only format. For future FAOBMB scientific meetings, it could be anticipated that hybrid formats may become the rule rather than the exception, so as to provide access to those who cannot travel. Nonetheless, future Conference and Congress organisers must keep their budgets in good shape so that registration fees cover the costs of mounting these scientific meetings, bearing in mind that there are considerable costs in mounting the virtual format, just as there are substantial costs for in‐person participants. It is thus likely that some sort of limited hybrid meetings may be considered for the future, with fee structures commensurate with the two types of participants (however, excessively large discounts for on‐line participants might have to be avoided).

**FIGURE 17:**
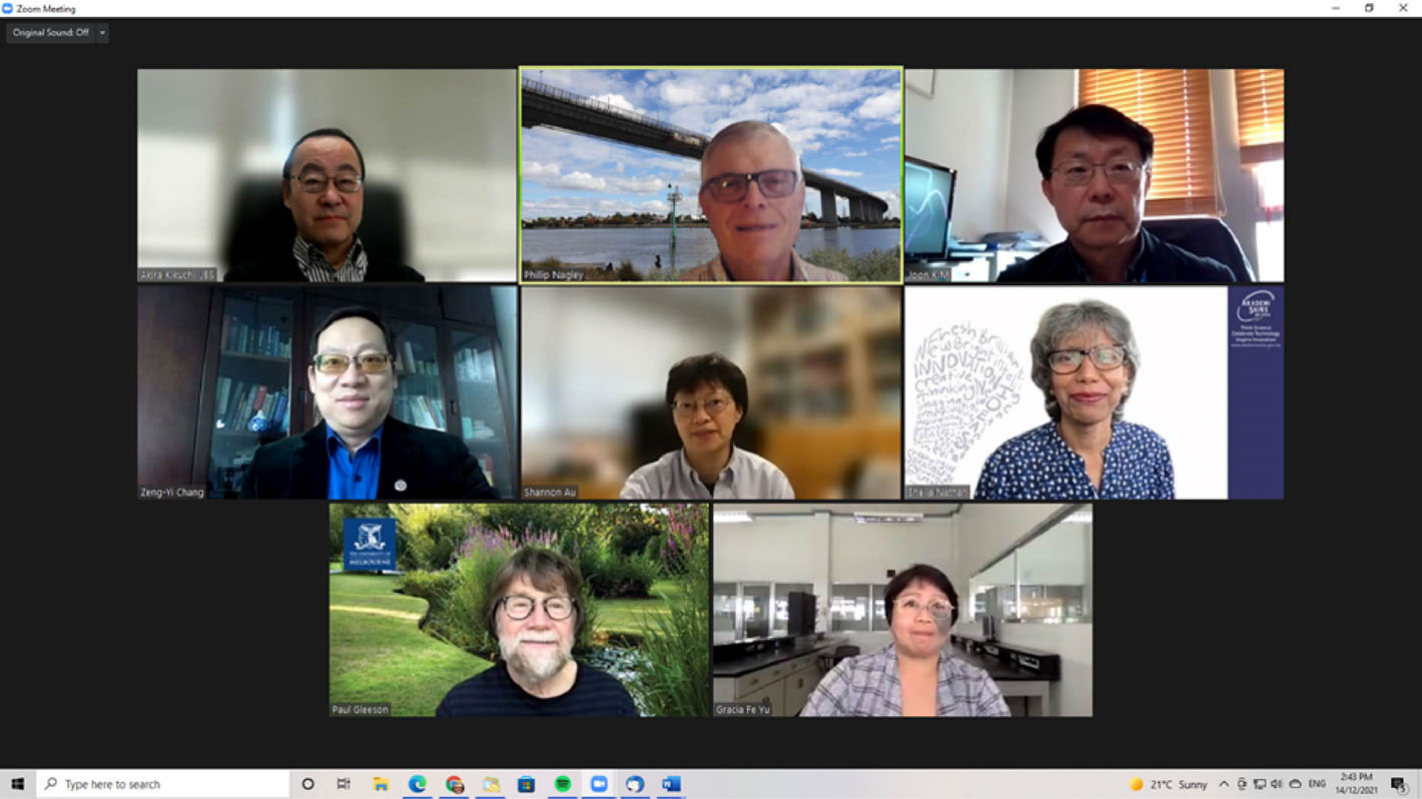
FAOBMB Executive Committee members participating in Zoom meeting held on 14 December 2021. Top row, from left: Akira Kikuchi (President), Phillip Nagley (Archivist, participating by invitation), Joon Kim (President‐Elect for 2022, participating by invitation); Middle row, from left: Zengyi Chang (Past‐President), Shannon Au (Treasurer), Sheila Nathan (Secretary General); Bottom row, from left: Paul Gleeson (Fellowships Chair), Grace Yu (Education Chair). Image captured as screenshot by P. Nagley

In addition, during the COVID‐19 pandemic the FAOBMB EC and Council meetings have so far all been held on‐line (Figure 17). The convenience afforded by the technologies such as Zoom to overcome the barriers of distance may be thought advantageous to save our time. However, when something is gained, something else may be lost, especially the personal contact and the ability that face‐to‐face meetings have for engendering productive discussions, especially on informal occasions.

Even though our virtual communications today can be used to overcome time and space, we still miss the good old ways of having face‐to‐face discussion with our colleagues in real locations with exposure to different cultures as happened so richly for the 27th FAOBMB Conference that took place barely 3 years ago in 2019. Furthermore, most biochemical and molecular biological discoveries come from real wet experiments in the laboratories. Our important job is to obtain solid and reproducible results by performing many such experiments (albeit supported by information technology that is now pervasive, and even applying novel approaches based on artificial intelligence that are on our doorstep). We are still facing difficult situations, but by joining forces to take on these tasks we should be able to overcome the obstacles. Beyond COVID‐19, we would combine both remote and real ways to progress policies and strategies of FAOBMB, which promote and strengthen research and education in various fields of biochemistry and molecular biology in our region and beyond.

## 13 CHALLENGES FOR THE FUTURE

FAOB(MB) was jointly founded in 1972 by three national biochemical societies and now has 20 Constituent Members. Various approaches have been continued to advance the academic community, stimulate the association of biochemists and molecular biologists in our region, and create networks that transcend barriers of ethnicity, culture, gender, and economic status. Over the years FAOBMB has developed steadily, promoting and strengthening research, and providing convivial platforms for dynamic interactions across the vast fields of biochemistry and molecular biology. With an age of 50 years, FAOBMB may be thought of as being in its ‘middle age’. It has certainly become a mature organisation, based on the sterling efforts and hard work of previous Presidents, Secretaries General, EC members, and others who have made contributions in various ways to the Federation.

FAOBMB is a family for all biochemists and molecular biologists in Asia and Oceania. The activities of the Federation need to reach as many of our colleagues as possible and make FAOBMB a balanced and harmonious community, in which the voice of each Constituent Member national society/group is heard, and all concerned are treated fairly and respectfully. By strengthening the IUBMB‐FAOBMB collaboration, fruitful progress and innovative developments in support of biochemistry and molecular biology all over the world can be confidently expected, even in the face of armed conflict in some regions.

Some strategic discussion on the future development of FAOBMB must be conducted, which includes the improvement of the following aspects: the Congresses and Conferences, the Awards, Education and Fellowships. Of course, continuing attention must always be paid by the leadership of the Federation to ensure a strong and stable financial position, and to maintain and develop our relationships with other regional federations and IUBMB. The continuing improvement of the quality of scientific education and research in Asia and Oceania is likewise very important. Success in these endeavours will, in turn, make the voice of FAOBMB heard locally, regionally, and more broadly in the world‐wide IUBMB community.

## CONFLICT OF INTEREST

The authors declare no conflict of interest.

## APPENDIX A

### A.1 Reflections from past office bearers

[*Presidents and Secretaries General, arranged in Alphabetical Order by Surname*].

#### A.1.1

##### A1.1 Professor Zengyi Chang

Professor, Peking University, Beijing, China.

(*President 2017–2019*)

The 50th anniversary of FAOBMB is certainly a momentous occasion for all the individuals associated with it in one way or another. My heartfelt congratulations!

Biochemistry and Molecular Biology have increasingly penetrated other fields, becoming the common language of all life science fields. Nonetheless, Biochemistry and Molecular Biology has its own unique mission. In the future, we need to explore the biomolecules in ways more quantitatively, rather than qualitatively; more in living cells, rather than in test tubes; more on the dynamic feature, rather than the static properties, etc. In this regard, Biochemistry and Molecular Biology will certainly remain a very active field for many more years to come.

FAOBMB represents a region of about 60% of the world population, being remarkably diversified in features such culture, language, political system, and status of scientific development. We need to be inclusive to bring all our potentialities into full play. We need to strive to make FAOBMB a family for all biochemists and molecular biologists in our area, trying to reach as many colleagues as possible and to make it a balanced and harmonious community, in which the voices of all types will be heard and respected. Together, we will improve the level of biochemistry and molecular biology research and education in our area, and in turn making a substantial contribution to the world community.

Finally, I wish FAOBMB to become the most active and influential among the Federations of IUBMB someday in the future.

##### A1.2 Professor John de Jersey

Emeritus Professor, The University of Queensland, Brisbane, Australia.

(*Secretary General*, *2006–2011*)

Like most Australian biochemists and molecular biologists in Australia during the second half of the twentieth century, my undergraduate, postgraduate, and postdoctoral education and training were focussed on Europe and North America, more specifically on the United Kingdom and the USA. This corresponded to a golden era for biochemistry and molecular biology and the related sciences of chemistry, genetics, cell biology and microbiology.

During my term as President of the Australian Society of Biochemistry and Molecular Biology, I served as the Australian delegate to the FAOBMB Council (2001–2003) which gave me an introduction to and appreciation of Asian biochemistry and molecular biology. It was clear that the member societies (and countries) varied greatly in the range and scope of the scientific endeavours they represented, ranging through highly developed, rapidly developing and still embryonic. I recognized that the FAOBMB had an important role to play in supporting education and research in biochemistry and molecular biology across the whole range of member Societies.

So, when Professor Bill Sawyer, of the University of Melbourne, Australia, and a former President of FAOBMB, asked if I would be interested in the position of Secretary General of FAOBMB, I gladly agreed to stand. I served in the role for two 3‐year terms (2006–2011) which proved fulfilling for me and covered a period of growth and development for the FAOBMB. The role was made pleasant and rewarding by the many office bearers and delegates that I worked with, in particular Hoon Eng Khoo, Masa Futai, Kyung‐soo Hahm, Kiyoshi Kita, Uhtaek Oh and Phillip Nagley.

The annual scientific meetings were the highlights of the year. During my 6 years as Secretary General, these meetings were in Kyoto, Seoul, Taipei, Shanghai, Melbourne, and Singapore, usually in concert with the annual meeting of the host Society and sometimes jointly with IUBMB. Very important components were the annual Young Scientists Program and Travel Awards, in conjunction with the scientific meeting.

During my time, we managed to institute a second Executive Meeting of FAOBMB each year, halfway between the annual conferences, and held either in the home institution of the President or in the city hosting the next scientific meeting. This allowed improved continuity of FAOBMB activities throughout the year. It was also recognized that FAOBMB members would benefit from an increased number of invited lectureships and awards for excellence, to be presented at the annual meeting. It is very pleasing to me to see that the FAOBMB continues to grow and support ongoing education and research in biochemistry and molecular biology in the Asian and Oceanian region.

##### A1.3 Professor Kiyoshi Fukui

Professor, University of Tokushima, Tokushima, Japan.

(*President 2014–2016*)

Western thought is suggested to emphasise forward‐looking approaches, whereas oriental thought represents a good combination of backward‐ and forward‐looking. We Orientals reflect the past to help foresee the future. In this context, it is a timely theme to update the history of FAOBMB at the time of the 50th anniversary of FAOBMB, to help the next generation of scientists in FAOBMB region to be reflective practitioners with a grand design in mind for our collectively bright future.

My service in FAOBMB started at the Executive Committee meeting in 2013, as President‐Elect. In April of that year, the Immediate Past‐President, Professor Futai, hosted an EC meeting at Morioka in Japan, followed by an FAOBMB Mini‐Symposium. One of the impressive events that Prof. Futai kindly arranged was the site‐visit to Rikuzentakata and Ofunato city, Iwate, the area affected severely by ‘Tsunami’ following the Great East Japan Earthquake in 2011. We deeply felt and realized the determination of people to recover from the disaster not only in their lives and the economy, but also in science.

We experienced the unfortunate cancellation of the 23rd FAOBMB Conference that was to be held in Dhaka, Bangladesh, because of the political unrest and civil disturbances in Bangladesh. In its place, a Special FAOBMB Symposium was successfully held in Singapore in 2013 with the great and intensive efforts and dedication of the Singapore Society for Biochemistry and Molecular Biology, and Siok Im Koh (FAOBMB Education Chair) in particular. I sincerely hope that someday in the near future FAOBMB will hold a scientific meeting in Bangladesh under the theme ‘Biochemistry & Molecular Biology in Socio‐economic Development’ as Prof. Qadri originally had planned for the 2013 Conference.

In 2014, we launched an award, the triennial FAOBMB Entrepreneurship Award, as an initiative to foster the entrepreneurial spirit among creative scientists in FAOBMB region, with the thoughtful dedication of Professor Andrew Wang and the great support of funding from the Yong‐Ling Foundation in Taiwan. We understand that entrepreneurship is becoming one of the most valuable elements in the present education programs at universities, which is now considered an important component of the so‐called ‘Innovation Ecosystem’.

For the 14th FAOBMB Congress at Hyderabad, India, in 2015 we experienced some difficulties in obtaining visa approval from the Indian government, and one of the EC members could not depart from his country because of the delay in visa issuance. Science has no borders, but scientists do occasionally encounter borders, largely not of their own making. Nevertheless, we should keep sending our message to the next generation of scientists how happy and fulfilled we feel to be as scientists, and to do our best to minimise such borders.

Our commitment and tremendous efforts to encourage recruitment of new members resulted in the establishment of new society in Sri Lanka, The College of Biochemists of Sri Lanka (CBSL) and its participation in FAOBMB community in 2016. In the Inaugural Symposium of CSBL, I had the honour of delivering the lecture referring to the ‘serendipity’ in my research and received the rewarding comments from the audience, after my mentioning the fairy tales of ‘Serendip’ (an ancient name for Sri Lanka).

Gender Equity (later referred to as Gender Equality) Policy was a major issue raised by Council members in 2014, which was followed by the development of the policy of FAOBMB. We are proud that our Executive Committee has been well organized based on this policy since my term in 2014.

The development of young researchers is the most important mission of science and technology promotion, and the need for quality assurance and standardization of research education in doctoral programs in each country will be increasingly required. From this perspective, it is important for senior researchers in the FAOBMB region to actively send their doctoral students and post‐doctoral fellows abroad to create a research environment in the region, which encourages friendly competition and creates a virtuous cycle of international academic and innovative brainpower. FAOBMB is strongly urged to play an important catalytic role in this effort.

I now sincerely hope our FAOBMB community will be able to focus on providing face‐to‐face experiences and networking through scientific meetings and other events, bringing us back again to pre‐COVID dynamics and restoring the fruitful times.

##### A1.4 Professor Masamitsu Futai

Formerly Professor, Iwate Medical University, Morioka, Japan.

(*President 2008–2010*)

Since this new History by Phillip Nagley, Jisnuson Svasti and Akira Kikuchi covers the most recent decades, I have not much to say about activities in my period as a President (2008–2010) or as Executive Committee member.

When I attended the council meeting in 1989, then Dr. T. Murachi was President, I was impressed by the critical discussion in very friendly atmosphere. Everybody was hoping strongly for the progress of biochemistry and molecular biology in the Asian and Oceanian region. This atmosphere has been kept for years as a good tradition in the Federation, and long‐term scientific friendships among members were established. In general, we can see the strong contribution of the Federation to the biological sciences.

I hesitate to mention anything retrospective, instead would like to discuss the new trend. Because of the pandemic, many meetings become remote including our bioenergetics conference last year. I could attend the first Zoom meeting of FAOBMB Council in 2021 as an observer. I felt that the members were not familiar with the new way of communication. However, Zoom is becoming easier since it is now used in most the educational and research institutes because of COVID‐19. Introducing the advantage of ‘remote’ communications, FAOBMB should make its home page more attractive for the Federation. Showing the videos of Symposium, Congress, or plenary lectures available from the home page would be valuable for many scientists and students. The Federation can also start web seminars or lecture series and show their videos of talks in the homepage. It would be timely to start a special committee for ‘Internet Communication’ in FAOBMB.

##### A1.5 Professor Kyung‐soo Hahm

Bioleaders Corporation, Daejeon, Republic of Korea.

Formerly Professor, Chosun University, Gwangju, South Korea.

(*President 2005–2007*)

The Korean Biochemical Society (currently called the Korean Society for Biochemistry and Molecular Biology; KSBMB) joined FAOB in 1973. I started to become involved in FAOBMB (FAOB then) as a general secretary for the 5th FAOB Congress which was held in Seoul, Korea in 1989, with 1400 participants from 35 countries. In 1993, FAOB became FAOBMB.

In 1999, the 6th IUBMB Conference and BioExpo'99 was held in Seoul, Korea and I served as the Secretary General of the conference. I served as a Korean delegate to FAOBMB from 1994 to 2002. In December 2003, I was elected as the President of FAOBMB, and that same year I attended the 10th FAOBMB Congress, which was held in Bangalore, India.

The next year, in November 2004, as the President‐elect I attended the 17th FAOBMB Symposium/second IUBMB Special Meeting/7th A‐IMBN Conference, which was held in Bangkok, Thailand. During the meeting, I organized a meeting inviting the previous FAOBMB presidents to listen to their opinions for the advancement of the FAOBMB.

In July 2005, as the President of FAOBMB I attended the 30th FEBS Congress/9th IUBMB Conference which was held in Budapest, Hungary. In November 2005, I attended 18th FAOBMB Symposium in Lahore, Pakistan, as the President. The catch phrase for the meeting was ‘Genomics and Proteomics in Health & Agriculture’. Because of the travel restrictions to Pakistan, not many foreign participants were present, except for a group of brave Australians. However, the Council meeting was held as scheduled without any problems.

In June 2006, the 11th FAOBMB Congress was held jointly with 20th IUBMB Congress in Kyoto, Japan. We were honoured to have the future Emperor of Japan (then Crown Prince) give us congratulatory remarks and to participate in a tea ceremony hosted by him, with dignitaries including myself. The FAOBMB Presidential Evening function was held with the generous support of the Shimadzu Corporation, at which were many invited dignitaries including former and present Presidents of FAOBMB and IUBMB. Also, a Shimadzu Evening was organized by the company for the entire participants in the Congress.

In May 2007, I attended the meeting of the Brazilian Society of Biochemistry and Molecular Biology (SBBq) XXXVI Annual Meeting in Salvador, Brazil. I participated as the President of FAOBMB in the IUBMB Executive Committee meeting. Also, in May 2007, the 19th FAOBMB Conference was held in Seoul with a catch phrase of ‘Science & Technology for the Integration of Life’. It was the last meeting for me as the President of FAOBMB. An FAOBMB Presidential Night was also organized with the generous support of the Shimadzu Corporation. A large FAOBMB banner was also prepared, but I am not sure where the banner is located now.

The 19th FAOBMB Conference was supported by many governmental or scientific organizations including the City of Seoul and the Korea Science Foundation. The scientific meeting consisted of plenary lectures, Special award lectures, a Women in Life Science Forum, and many symposia including a Korea‐Sweden Symposium and several workshops. There was also a large trade exhibition termed BioExpo. All these events were held in the extensive Coex Convention & Exhibition Center located in the Gangnam district of Seoul.

As the Past‐President, I attended the 20th FAOBMB Conference in Taipei, China in 2008 and the 21st IUBMB/12th FAOBMB Congress of Biochemistry and Molecular Biology in 2009 in Shanghai, China.

##### A1.6 Professor Hoon‐Eng Khoo

Formerly Professor, National University of Singapore, Singapore.

(*Secretary General, 2000–2005*)

The period spanning 1999–2011, when I was Secretary General and later Chair of the Education Committee of FAOBMB, was an exciting one. FAOBMB expanded its impact and promoted biochemistry and molecular biology in our region and beyond. We paid particular attention to having special symposia on Education at FAOBMB‐sponsored meetings. Examples include workshops on Biochemical Education sponsored jointly by the IUBMB, FAOBMB and the host national Society or Group of Biochemistry and Molecular Biology, which allowed our young members to network and explore collaborative opportunities among themselves and with the host institutions. The Young Scientist Programme (YSP) organised from time to time allowed sharing of their research and opportunities for professional development. Such networking was facilitated by senior scientists working in related fields that were non‐university/non‐research, who were also invited to take part of such YSP events. Workshops on the latest research techniques were conducted. For example, in May 2011, FAOBMB participated in sponsorship of a ‘Workshop on Functional Genomics and Structural Biology’ in Malaysia. Travel fellowships allowed young scientists from less‐developed economies to attend FAOBMB conferences.

In recognition of outstanding biochemists and molecular biologists in our region, the FAOBMB Medal (later called the FAOBMB Award for Research Excellence) was first awarded at the FAOBMB Conference held in Singapore in October 2011, with a generous sponsorship organised by the Society for Biochemistry and Molecular Biology located in Taipei, China. The next year, the FAOBMB Education Award was inaugurated to recognize excellent contributions to biochemistry and molecular biology education. FAOBMB Exchange Fellowships for early career biochemists and molecular biologists were also introduced. To ensure better communication among members, a website of professional quality was set up in 2011, hosted by Academia Sinica in Taipei. Working closely with the IUBMB Education Chair at the time (Susan Hamilton, a previous FAOBMB Education Chair), we managed to jointly sponsor/fund education workshops in our region.

Apart from these research and educational activities, I am also proud of FAOBMB for supporting member organisations in need. For example, after the devastating typhoon Nargis hit Myanmar in 2008, FAOBMB contributed funds to the Myanmar Biochemists Group to support their recovery. From 2011 to 2013, a donation was made by FAOBMB to a start‐up university, the Asian University for Women (AUW) in Bangladesh. This generous donation allowed AUW to purchase equipment and start courses in biochemistry and molecular biology which enabled their students to progress to graduate schools and support other students majoring in their Public Health and Environmental Sciences programmes.

Overall, I believe FAOBMB has made important contributions to the development and promotion of biochemistry and molecular biology activities in the Asia Pacific region and beyond, by collaborating closely with IUBMB and other similar organisations worldwide.

##### A1.7 Professor Kay Hoon Lee

Associate Professor, National University of Singapore, Singapore (*Deceased 2003*).

(*Secretary General 1988–1993*)

*Reprinted (with minor editorial corrections by the authors PN and AK) from*: *Addendum to the Report of the Secretary‐General, Agenda for FAOBMB Council (1993)*.

Being the last report from me as the Secretary‐General of the FAOB Inc., I would like to take this opportunity to thank you all very much for the support and co‐operation you have given me since I took office from January 1988. Without such support and co‐operation, it would have been impossible for me to run the office of the Secretary General. My special thanks go to Professor N. V. Bhagavan and Professor J. Svasti, whom I have known and worked with since I got involved in the FAOB, and to Professor Y. Anraku, whom I got to know when he became a member of the Executive Committee in January this year. They have been a great help in my running of the office of the Secretary General.

I would also like to take this opportunity to extend my sincere apologies to anyone whom I had inadvertently offended through my ways of handling the affairs of the FAOB. I have no intention of offending anyone. At times I might have pushed hard to get things done. All was done with the interest of the FAOB at heart. Up to date I still feel very strongly that the FAOB must move fast or else it will be left behind by other similar organisations. We should adopt the attitude that if things other organisations can do, we not only can do but can do better, so long we have the will to do so. The kind of the FAOB we want depends on us in the FAOB. It could be either an organisation that takes things as they come or a very dynamic one that lights up like a beacon showing the ways in the science of biochemistry in the region as well as the world. Together we can make this Federation one of the most respected scientific organisations on earth.

Much has happened since I took office in 1988. The FAOB Statutes were amended in 1989 since its last amendment in 1978. I was given the honour of revising and re‐writing of the Statutes. The new amended Statutes incorporated a new category of membership, namely, Special Membership. This Clause enables the FAOB to invite companies to become Special Members. The amended Statutes were subsequently used to form the Statement of Purposes and Rules of the FAOB when it was incorporated in the State of Victoria, Australia.

Until its incorporation on 8 December 1992, the FAOB had existed since its founding in 1972 without any legal status. We had looked hard at possible places for an official registered address for several years without success. The incorporation of the FAOB in the State of Victoria was the result of a discussion at an informal social gathering hosted by Professor Anthony W. Linnane (Australia) during the 15th IUBMB Congress in Jerusalem in 1991. If I am not wrong, Professor N. V. Bhagavan brought up the issue during that informal gathering. The other point discussed was to get tax‐exemption for the Federation.

Professor Linnane suggested the possibility of incorporating the FAOB in Victoria, Australia and promised to assist in whatever possible. Through him and Dr. Barry Preston we managed to get Mr. McQuillan (a Melbourne lawyer) to proceed with the incorporation. After much exchange of correspondence among various parties, and about one and a half year since the meeting in Jerusalem, the FAOB finally found its home in Victoria, Australia. Not only did we manage to have the FAOB incorporated in Australia but also, we were able to get tax‐exemption status for the FAOB from the Australian Taxation Office. I would like to record our special thanks to Professor Linnane, Dr. Barry Preston and Mr. McQuillan for assisting in getting the FAOB incorporated in Australia.

The present logo, submitted by Professor Sang‐Sup Lee of Korea, was adopted at the Council meeting in Seoul in 1989. We had to change the previous logo of a weather cock to the present one because the FAOB has no exclusive right of use of the earlier logo.

The saddest news during my term of office was the death of Professor Takashi Murachi. As far as I can recall, late Takashi had not missed any of the FAOB meetings. With his death, the FAOB has lost one of its strongest advocates. I am glad that Takashi Murachi lives on in the FAOB with the establishment of the Murachi Lectureship. The initial sum of $20,000 to establish the Murachi Memorial Lectureship Endowment Fund was generously donated by Mrs. Murachi.

During my term of office, two new members were admitted to the FAOB. Myanmar Biochemists Group became a member of the FAOB on 5 May 1990, and the Biochemists Group of Viet Nam Universities and Pedagogical Institutes (BGVN‐UPI) became the 18th member on 2 September 1993. It is hoped that more societies or groups of biochemists in the region will join as members of the FAOB. Eventually, the FAOB should be *the* organisation for the biochemists in the Asian and Oceanian region and to serve their interest.

It is heartening to note that the FAOB Congresses and Symposia are recognised and well‐known events in the scientific calendar. Their scientific standard is comparable with that of other international scientific meetings held elsewhere. Let us keep up with the good work.

The FAOB still faces the problem of communication, although there have been improvements since I took over the office of the Secretary General. Often there was no response from some delegates when reply was requested. Professor O'Sullivan, Editor of the FAOB News and Editor of the newly established FAOB Bulletin, has always faced the great problem of getting news items from the respective members. Appeal after appeal has been made at Council meetings. Somehow, news is still hard to come by. Therefore, more effort needs to be put in by members of the FAOB to provide news or short write‐ups for the FAOB Bulletin. I must say that Professor O'Sullivan has done a splendid job in having the FAOB News published regularly despite the difficulty which he has encountered in getting news items from the members.

It has been my hope that the FAOB Journal could take off the ground during my term of office. The journal issue was discussed from the day the FAOB was established. Looking at the minutes of earlier Council meetings, I realised that there was little follow up on the issue after each Council meeting. This could be due to great changes in the delegates to the FAOB Council in the early days of the FAOB. In 1988, I brought up the issue again for discussion at the Council meeting in Kuala Lumpur, and advocated strongly that it would be beneficial, both scientifically and financially, for the FAOB to have its own scientific journal. To my disappointment I found support only from a few of the Council members present. I remember there were many arguments against the publication. However, at the end of the day, I managed to win some over and the Council decided to set up a sub‐committee to look into the issue. In the subsequent Council meetings, more Council members were convinced that the FAOB should publish a scientific journal. Sub‐committees were further formed to look into more details about the journal. I must say that it was a tough job to do. In 1989, I managed to convince a publisher in Singapore to finance the publication. To my great disappointment and regret, the Council was not positive enough to take up the offer at that time and in the year that followed. Nevertheless, it is good news that the Journal Sub‐Committee chaired by the Managing Editor Designate, Professor Richard Guillory, is working hard on the FAOB Journal. Certainly, it is even more heartening to see the support given by the Japanese Biochemical Society to the publication. Professor I. Yamashina, a prominent scientist from Japan, has agreed to be the Chief Editor Designate. Undoubtedly, the journal is in excellent hands. It is my hope that once the FAOB decides to go ahead with the publication of the journal, all of us in the FAOB should give it our strongest support possible. It is our journal, our intellectual property, and we should be proud to be associated with it. I hope to see the birth of this new journal soon.

Finally, once again my grateful thanks to you all for the wonderful friendship which you have given me. I will treasure it in the rest of my life.

##### A1.8 Professor Phillip Nagley

Emeritus Professor, Monash University, Clayton, Melbourne, Australia.

(*Secretary General 2012–2017*)

I attended two FAOBMB scientific meetings in the 1990s, the first being the 7th FAOBMB Congress in Sydney in 1995, which was held alongside the regular Annual Meeting of the Australian Society for Biochemistry and Molecular Biology (ASBMB) that year. My second was the 13th FAOBMB Symposium in Manila in 1997, to which I had been invited by my colleague Yau‐Huei Wei from Taipei, to participate in a session on mitochondria, my field of research. Little did I know it then but a decade later my serious involvement in FAOBMB would begin.

My own interest in the international aspects of scientific organisation was kindled in the early 2000s. I was to become President of ASBMB in 2004 and, as President‐Elect, I represented Australia at the General Assembly of IUBMB in Montreal, Canada in 2003. From this experience, I began to see the importance of International Bodies in the leadership of scientific research and publishing, together with well‐designed training and educational policies, not only at the globally international level but also at the regional level where Federations such as FEBS and FAOBMB were playing such an important role.

It was my involvement in the planning of the OzBio2010 Conference to take place in Melbourne in 2010 that brought me into close contact with FAOBMB. OzBio2010 was the nickname for the 21st FAOBMB Conference, to be jointly held as the 12th IUBMB Conference, together with ASBMB and national societies of other disciplines including Cell and Developmental Biology, and Plant Sciences. My responsibilities were to be focussed on international liaison, as well as organising the YSP event that was to take place in association with the Conference in 2010.

Accordingly, I presented the proposals for the OzBio2010 Conference first to IUBMB, then to FAOBMB in 2005. I attended the meeting of FAOBMB Council held in Lahore, Pakistan, in association with the 18th FAOBMB Symposium. My formative experiences in Lahore included meeting members of FAOBMB EC and other Council delegates, enjoying the wonderful hospitality provided by our hosts led by Waheed Akhtar, observing the scientific and technical endeavours of the local biochemical community in Pakistan, and witnessing the amazing cultural events organised in association with the Conference. All these enabled me to appreciate the value and importance of FAOBMB in bringing regional scientists together with shared interests in biochemistry and molecular biology.

For the next few years, I attended every meeting of FAOBMB Council as delegate of ASBMB to report progress on planning OzBio2010. John de Jersey (then Secretary General) arranged for me to attend meetings of FAOBMB EC in 2010 and 2011, initially to brief EC on detailed planning for OzBio2010, and later for me to be drawn into FAOBMB affairs with greater engagement. In early 2011, John told me that he was in the last year of his term of office and asked me if I would like to stand for the position of Secretary General, which I agreed to do.

In 2012, during my first year as Secretary General, the State of Victoria (where FAOBMB is registered as an Incorporated Association) passed a new Act of Parliament setting out new rules and regulations for Incorporated Associations. To accommodate this new Act, the Rules of FAOBMB had to be restructured completely to fit in with the new requirements. Therefore, I had to rewrite the Rules of FAOBMB and to make extensive revisions and updates to the Standing Orders, ready for the consideration of Council at its meeting in 2013 (which eventually took place in Singapore in December of that year). After amendments were made at Council in 2013, the amended Rules and Standing Orders were approved at Council in 2014, in Taipei. Updates and improvements to the Standing Orders have been made on a few occasions since then, some after 2017 when I finished as Secretary General and became Archivist in 2018. I have been working with current Secretary General, Sheila Nathan, to assist with these important constitutional documents of the Federation.

As Archivist in 2018, I inspected closely the large boxes of FAOB(MB) documents that John de Jersey had passed on to me at the end of 2011. To my dismay, I realised that the set of documents was incomplete (no fault of John, I should add). There was barely anything from the foundation of FAOB in 1972 until 1988 (except for copies of the signed document agreeing to establish FAOB and the first Statutes of FAOB). Printed and bound copies of all Council documents from 1988 until 2009 were provided, with the unfortunate exceptions that such Council documents from 1997 to 1999 were missing. After 2009, all documents for Council and EC were in electronic form, to which I had access. Fortunately, Jisnuson Svasti had kept many records of FAOB from the mid‐1970s to the early 1990s, including Council Minutes and some Newsletters/Bulletins of the Federation. Jisnuson also has been most generous in providing photographs from his period with FAOB, which together with photos that I have accumulated during my association with FAOBMB, cover much of the entire 50‐year history. As Archivist, my aim is to place as many archival documents as possible onto an on‐line file storage repository.

Rather than reflect on all the experiences I have had over the past three decades with FAOBMB, including the interesting places I have travelled to, the wonderful people I have met and the many friendships that I have made, I shall mention just two further points. First concerns the webpage, which I was able to have converted in 2016 into a user‐friendly format in WordPress. The new webpage is based on the excellent layouts and content that Andrew Wang, Shan‐Chi Ku and their colleagues at Academia Sinica in Taipei had first built in 2010. The webpage is one very important node of communication for the FAOBMB community and is the window to the world of FAOBMB. It is a real pleasure for me to be able to keep the webpage up to date with news and information, as well as maintaining its function as the repository for all users of key documentation for many of the activities of FAOBMB. In this task, of course, I am assisting the Secretary General, who has the statutory responsibility of maintaining the webpage and being responsible for its content.

Second concerns the vitality and longevity of FAOBMB. It is my opinion that it is the organisational structure of FAOBMB (involving members of the EC working in partnership with the Constituent Members through their delegates to Council) and its openness in recording the details of its deliberations and policy making, which have made the Federation robust and productive over the past five decades. Careful and responsible management of the finances and clear visions for the future from its leadership have enabled the Federation to make its mark as the key regional organisation for Biochemistry and Molecular Biology in Asia and Oceania.

##### A1.9 Professor William H. Sawyer

Emeritus Professor, The University of Melbourne, Victoria, Australia.

(*President 1999–2001*)

I have followed the FAOBMB progress with interest and have been impressed with the continued vitality of the organisation. I look forward to the 50th Anniversary of the Federation.

Two things come to mind concerning my presidency—one good and one not so good.

I was surprised to discover that students in a number of countries did not own a biochemistry text. Some had no access to a library. Bruce Stone and I arranged for the ASBMB (Australia) community to save old texts when a new edition was issued. Bruce arranged a generous price for air transport with Qantas, and we were able to distribute these texts to deserving member countries at the next FAOBMB meeting.

The saddest thing was the demise of the FAOBMB Journal, which was first launched in 1997. For many years the publisher subsidised the publication of the *Journal of Biochemistry, Molecular Biology and Biophysics* but FAOBMB was never in a position to assist with funding. The new publisher, who took over the Journal in 2001, refused to continue with the subsidy. As I recall, the Japanese Biochemical Society offered to underwrite or guarantee the sale of a certain number of subscriptions to the FAOBMB Journal. This was not enough for the new publisher. Our extensive pleading was of no use, and we were just one of a number of journals that were terminated. The FAOBMB Journal was instrumental in helping young scientists from the poorer countries to succeed in print. In hindsight, the decline of the printed FAOBMB Journal was probably inevitable. At the time we had extensive discussions, but little experience, in online publishing.

##### A1.10 Professor Jisnuson Svasti

Emeritus Professor, Mahidol University and Chulabhorn Research Institute, Bangkok.

(*Treasurer, 1980–1986 and President, 1990–1992*)

I was just less than 30 years old when I first became involved with FAOB in 1976, after receiving an IUB Travel Fellowship to the 10th IUB Congress in Hamburg. There, FAOB President, Anthony W. Linnane (Australia) called an FAOB Council Meeting on 30th July 1976, attended by Executive Committee, Takashi Murachi (Japan) and N. R. Mougdal (India), as well as five Council Members. I represented the Biochemical Section, Science Society of Thailand (BMB Thailand) and felt honoured to act as Recorder to help Murachi with the Minutes. Then, FAOB had only 12 Constituent Members, and financial reserves of US$ 3,762. Most importantly, the original FAOB Constitution was amended to expand the Executive Committee to four members, namely President, President‐Elect or Immediate Past President, Secretary‐General and Treasurer. Everything went smoothly, since Linnane previously arranged an Executive/Council Meeting in Bangkok during 26–28 February 1976.

In 1977, Mervyn G. Smith (New Zealand) and Serene Vimokesant (Thailand) were elected Secretary‐General and Treasurer of FAOB, respectively, and Kazutomo Imahori (Japan) became President, FAOB in 1978. I attended the 2nd FAOB Symposium in 1979 and accompanied Vimokesant, my Department Chairman, to the 2nd FAOB Congress in Bangalore, 1980. At the Council Meeting, I was surprised to be unanimously elected as the next Treasurer of FAOB. Smith also resigned, and Fyfe L. Bygrave (Australia) was appointed Secretary General. Enjoying this experience of interacting with biochemists in the region, I attended all the FAOB(MB) Symposia and Congresses from 1979 to 1995.

When Osamu Hayaishi (Japan) became President, FAOB (1981–1983), he invited Fyfe Bygrave and me to meet with him, Imahori, Kunio Yagi (IUB representative), and senior members of the Japanese Biochemical Society (JBS) in Tokyo during 18–19 March 1981. As a former IUB President, Hayaishi had an inspiring vision of how FAOB should develop and was fully supported, both in principle and financially by JBS. These development plans required more budget, so FAOB Council approved a new subscription system in Bali, June 1981, where each Constituent Member had equal vote but could select their subscription rate as: $0.25, $0.50 or $1.00 per society member. This significantly increased subscription income. Then, the FAOB Council Meeting at the 12th IUB Congress, Perth, Australia, 1982, approved a donation by Suntory Company to fund a JBS Lecture.

In 1983, Thailand hosted the 3rd FAOB Congress, which helped to make biochemistry in Thailand more visible worldwide. Hayaishi delivered the first JBS Lecture, later named the Osamu Hayaishi Lecture. Re‐elected Treasurer for 1984–1986, I was glad that FAOB became a Regional Associated Organization of IUB in 1983, since IUB(MB) provided important financial support. Then, Murachi became President, FAOB for 1987–1989, and I was again surprised to be nominated and elected to be President of FAOB for 1990–1992. As President‐Elect, I attended the 5th FAOB Congress in Seoul in 1989, where the previous logo of weathercock devised by Kunio Yagi, was replaced by a new design, reflecting the dynamic and continual flow of information among members; this design is still used today after modifying FAOB to FAOBMB. The Constitution was also revised to add a new category of Special Member for companies.

When I became President, FAOB for 1990–1992, I appreciated assistance and advice from Kay Hoon Lee (Secretary‐General) and Nadhipuram V. Bhagavan (Treasurer) in addressing the major problems of FAOB, namely finance and communication. The *FAOB News* (later *FAOBMB Bulletin*) gained strength when William J. O'Sullivan (Australia) became Editor in 1984 for 10 years or more. Still, I continued Murachi's practice of writing to Council Delegates. However, my monthly *Letter from the President* did not just provide news, but also tried to instil a sense of unity and purpose for FAOB, based on the concept that ‘*FAOB exists for and exists through the cooperation of member societies*’. Thus, we should all ‘*Help FAOB Help Us*’. When Murachi, who served FAOB since its foundation, suddenly passed away in 1990, donations were made by Mrs. Murachi and scientists worldwide to endow the Murachi Memorial Lecture. Later in 1993, Kunio Yagi and I separately donated funds to establish the Kunio Yagi Lecture and Jisnuson Svasti Lecture, respectively.

It was a shock that the 8th FAOB Symposium, Bangladesh in 1990 had to be postponed at the last moment due to political disturbances, so I held a Council Meeting in Bangkok on 25 March 1991 to address important issues. However, since financial support had already been provided, the Bangladesh Symposium was reconvened on 11–13 June 1991, encouraging biochemistry locally. I also took over Murachi's role in recruiting companies donating US$ 1,000 per year to become Special Members, with the first two companies receiving plaques at the 9th FAOB Symposium in Hong Kong, 1991. By 1994, there were 20 Special Members, many from Japan, providing another important income source.

Finally, the 6th FAOB Congress in Shanghai, 16–21 November 1992, was the culmination of my activities as President and was attended by 500 participants (46% overseas) plus 200 observers. The Opening Lecture was the Inaugural Murachi Memorial Lecture on 16 November 1992, held as a tribute to a founder who helped FAOB develop, with a plaque of honour being presented to Mrs. Etsuko Murachi. I also held a President's cocktail party for Council Delegates, Organizing Committee, Invited Speakers, and VIPs. Then, the 20th Anniversary of FAOB was celebrated with a Cultural Night Reception held by the host society. In addition, there was an Exhibition Booth showing historical events of FAOB, based on information from Constituent Member societies and historical comments from former Presidents. This included a photograph provided by Edwin C. Webb (Australia) of first Executive Committee, recalling he, Murachi and Mougdal having had initial discussions in Bangalore about a regional biochemical association, 2 years before the foundation of FAOB.

Most importantly, the Incorporation of FAOB as a tax‐exempt organization in Victoria, Australia, was approved by Council. This incorporation was arranged within 15 months from our first communication with Alan McQuillan, a lawyer recommended by Barry Preston in Melbourne. Official registration is essential for effective operation of any society, so FAOB became officially incorporated on 8 December 1992, with William H. Sawyer (Australia) as Public Officer: FAOB was later renamed FAOBMB in 1993.

I continued as Immediate Past President for 2 years and served as Chair of the 11th FAOBMB Symposium in Bangkok in 1994. Lastly, I was glad to chair the first Jisnuson Svasti Lecture, delivered by Linnane at the 7th FAOBMB Congress, 1995 in Sydney, Australia, since Linnane had introduced me to FAOB(MB). Thus, this lecture was a fitting end to my formal involvement with FAOB(MB) in the early days. However, I have continued to act as Advisor to FAOBMB scientific meetings held in Thailand, such as those held in 2004 and 2012, as well as the upcoming meeting in 2023, because FAOBMB is still important to me.

##### A1.11 Professor Andrew H.J. Wang

Emeritus Professor, Academia Sinica, Taipei, Taiwan.

(*President 2011–2013*)

In the year 2000, I retired from the University of Illinois Champaign‐Urbana, USA, and accepted the position of Director of the Institute of Biological Chemistry, Academia Sinica, in Taipei, Taiwan. That was the turning point for me to connect with the public activities associated with field of biochemistry and molecular biology. I was elected as the President of Taiwan Society of Biochemistry and Molecular Biology (TSBMB) in 2001, which gave me the opportunity of learning how to lead a scientific society. Our tasks included the need to organize annual conferences, recruit new members, raise funds to support the various activities, and expand the training opportunities for young students.

My first responsibility for involvement in an international event organized by TSBMB was to run the 16th FAOBMB Symposium in 2002, which was held on the campus of Academia Sinica (the venue having been secured by my predecessors in TSBMB). One of the things that stuck in my memory of that Symposium was the Executive Committee (EC) having an intense discussion on the fate of the FAOBMB Journal, whose Editor‐in‐chief was Richard Guillory, a close colleague of Professor Ram Bhagavan from Hawaii (Ram was a long‐time supporter of FAOBMB, who passed away in June 2018). After much deliberation, EC had to accept the publisher's decision to terminate the journal. The situation illustrated the difficulty of running a regional journal without the involvement of a commercial publisher. The following year, the 10th FAOBMB Congress was held in Bangalore, India in 2003. TSBMB had a team of biochemists attending that congress in 2003, including some invited speakers from our society. That was my first ever visit to India. I was impressed by the very good organization of the Congress by our Indian hosts, complete with wonderful cultural activities of traditional Indian music and dance.

I attended the 17th FAOBMB Symposium held in Bangkok, Thailand in 2004 (designated as the second IUBMB Special Meeting). During the cruise ship tour on the Chao Phraya River in Bangkok, I had a chance to chat with Professor Bill Sawyer, who was the President of FAOBMB during 1999–2001. I remembered he had retired from his post at the University of Melbourne, and he was running a vineyard producing some nice Australian wines. We talked about the history of FAOBMB, and he encouraged me to be more engaged with FAOBMB, which inspired me to seek the opportunity to serve the Federation. Later, I became the President‐elect in 2010 and served as the President in 2011–2013.

During my presidency, I looked for financial support from private foundations to initiate the establishment of new awards for the federation. Although the FAOBMB already had several named lectureships, the infusion of sustained new awards to highly achieving biochemists and molecular biologists from our region was considered valuable to our Federation. With the initial funding support from Yong‐Ling Foundation in Taiwan (a foundation funded by Terry Kuo, the Chairman of FOXCONN), we established three awards: Research Excellence Award, Education Award, and Entrepreneurship Award. I am grateful to Professor John de Jersey, Secretary General of FAOBMB, for preparing the documentation for the Research Excellence Award, including drafting the relevant new Standing Orders, the Guidelines and the Nomination Form. The first Award for Research Excellence was bestowed on Professor Cheng‐Wen Wu from Taiwan and the award was given during the 22nd FAOBMB Conference in Singapore, 2011. The details about those awards can be found in the present FAOBMB website at www.faobmb.com. After 3 years, the funding from Yong‐Ling was ended and the financial support for the awards was transferred to the Biochemical Technology Education Foundation (BTEF, a non‐profit foundation set up by me in Taiwan) since then up to now. Every year, BTEF pays for the award prize and provides an inscribed plaque in honour of each awardee. I retired as FAOBMB Past‐President in 2015. The FAOBMB Executive Committee and Council held a farewell luncheon for me during the 14th FAOBMB Congress in Hyderabad, India and I passed the presidential baton to Professor Kiyoshi Fukui.

During my term of the Presidency time (2010–2015), a few things are worthy of mention. On March 11, 2011 a gigantic earthquake (magnitude 9 on the Richter Scale) occurred near the north‐eastern coast of Japan. The accompanying huge tidal wave (Tsunami) devastated that area, thousands of people lost their lives, many buildings were damaged (including the Fukushima nuclear powerplant and the Sendai airport), and there were severe economic losses. Professor Masamitsu Futai from Iwate Medical University was the Past‐President of FAOBMB during that catastrophe. His university in Morioka was also damaged. I do not remember whether the famous rock‐breaking cherry tree nearby was affected or not. Professor Futai and his team from Iwate Medical University, in collaboration with the FAOBMB EC, organized a mini‐symposium in his university in April 2013. A tour to the disaster area was arranged. We witnessed first‐hand how serious was that earthquake of 11 March 2011. Although by that time much of the damaged buildings and landscape had been cleaned up and recovered, the striking images still were shocking to us. We used the visit to pay tribute to our dear FAOBMB friends, especially Professor Futai and his colleagues in Morioka.

In the EC and Council, the Secretary General and the Treasurer are two important positions. During my term as President, Professor John de Jersey from the University of Queensland was the Secretary General. He was about to complete his second 3‐year term at the end of 2011, and he suggested that his Australian colleague from Monash University, Professor Phillip Nagley, could succeed him. The Secretary General position requires high proficiency in English and excellent organizational skills to manage the wide variety of matters concerning the Federation. Phillip, John and I decided to visit the FAOBMB Treasurer, Professor Uhtaek Oh from Seoul National University (2008–2013), to get familiar with one another. Not only through the EC meeting held in Seoul in May 2011, but also through many informal discussions, this turned out to be an important gathering. It gave us all a better view of our finances and the status of various official documents. In the subsequent years, Phillip has spent great effort in getting all kinds of information and processes of the Federation in order. Uhtaek also made the financial matters more transparent, than had previously been the case, so that when Professor Piamsook Pongsawasdi from Bangkok succeeded him as Treasurer in 2014, it was a very smooth transition. I would like to mention that during our visit to Seoul, Uhtaek treated us with a nice Korean‐style BBQ and arranged a very special tour for us of the famous DMZ line at the 38th Parallel. The underground tunnel built by the North Koreans, initially for military purpose, is now a tourist site.

During my time as Past‐President, I organized (as conference chair) the 15th IUBMB‐24th FAOBMB‐TSBMB International Conference (21–26 October 2014) in Academia Sinica, Taipei. The event was nicely reported by Professor Leann Tilley (available on the FAOBMB website). Besides the usual plenary and invited scientific talks, there were several activities worthy of mention. We organized an ‘Education Symposium on Writing in Science' delivered by Dr. AndreAna Pena from the Institute of Molecular Biology, Academia Sinica and Professor Phillip Nagley. The symposium was a huge success, attended by many young scientists who were eager to learn the skills. We also organized a special session on entrepreneurs delivered by Professor Paul Schimmel. Another highlight was the ‘Youngs Meet Laureates’ career development session, which also drew many young scientists to attend. All in all, it was a successful conference, and we controlled the expenditure costs very effectively, resulting in a surplus which was reimbursed to three sponsoring organizations: IUBMB, FAOBMB and TSBMB.

The IUBMB EC members also held their meeting during the Conference, and they witnessed the success of the conference. I recall that Professor Mike Walsh (IUBMB General Secretary) and Professor Joan Guinovart (IUBMB President) approached me to see whether I might consider the Presidency of IUBMB. After much soul‐searching, I accepted the invitation to send in my name for consideration. I was elected as President‐elect of IUBMB during the 23rd IUBMB Congress held in Foz do Iguaçu, Brazil in 2015. I became the President at the end of the 24th IUBMB Congress in Seoul, Korea in 2018.

During my tenure as the IUBMB presidency, I continue to promote the involvement of FAOBMB in the international biochemistry arena. Moreover, I continued to attend all the FAOBMB activities after I stepped down as Past‐President of FAOBMB. Unfortunately, the COVID‐19 pandemic disrupts almost all the international conferences. FAOBMB is no exception. The 28th FAOBMB Conference that was to be held in Colombo, Sri Lanka, in June 2020 was cancelled and the 16th FAOBMB Congress that was scheduled for Christchurch, New Zealand was held as a completely virtual meeting (and certainly very successfully, despite the many difficulties in organizing such a meeting entirely on‐line—sincere congratulations are due to Wayne Patrick and his NZSBMB colleagues for doing that job so well). With the Omicron strain of COVID‐19 still rampant around the world, all we can hope is the vaccination coverage will be sufficient, or enough herd immunity developed, to allow the national borders to open more widely. When such days come, much less restricted international travel will be possible, so we will be able to see our FAOBMB friends in person again. I eagerly look forward to that!
